# Bispecific antibody vesicles: A multifunctional bioactive drug delivery platform for the treatment of *Pseudomonas aeruginosa* infection

**DOI:** 10.1016/j.ajps.2026.101162

**Published:** 2026-05-17

**Authors:** Jiaxin Ma, Zihao Teng, Xuqi Peng, Yanyin Wang, Linyu Ding, Yijia Xie, Wenhui Huang, Qiuyue Long, Jianzhong Zhang, Lai Jiang, Gang Liu

**Affiliations:** a State Key Laboratory of Vaccines for Infectious Diseases, Xiang An Biomedicine Laboratory, National Innovation Platform for Industry-Education Integration in Vaccine Research, Fujian Engineering Research Center of Molecular Theranostic Technology, Center for Molecular Imaging and Translational Medicine, School of Public Health, Xiamen University, Xiamen 361102, China; b School of Pharmaceutical Sciences, Xiamen University, Xiamen 361102, China; c School of Medicine, Xiamen University, Xiamen 361102, China; d Department of Neurological Surgery, Feinberg School of Medicine, Northwestern University, Chicago, Illinois, USA; e School of Pharmaceutical Sciences, Zhejiang Chinese Medical University, Hangzhou 311402, China

**Keywords:** Bispecific antibody-presented membrane vesicles, Biofilm, *P. aeruginosa*-induced pneumonia, Nano antibiotic, Antibody-antibiotic therapy

## Abstract

*Pseudomonas aeruginosa* (*P. aeruginosa*) is a major pulmonary pathogen that establishes chronic infection through Psl polysaccharide-mediated adhesion and biofilm formation, while accelerating acute disease via toxin injection through the type III secretion system (T3SS) PcrV protein. Conventional small-molecule antibiotics show poor target specificity, limited biofilm penetration and insufficient toxin neutralization, thereby compromising therapeutic efficacy. Here, we developed a bioactive nano delivery system that integrates a bispecific antibody (BsAb) with a nano-antibiotic for *P. aeruginosa* infection. Specifically, an anti-Psl single-chain variable fragment (scFv) was linked with an anti-PcrV monoclonal antibody (mAb) to generate the BsAb, which was subsequently displayed on cellular outer membranes and harvested as nanoscale membrane vesicles displaying BsAb (BsAb-MVs). Gentamicin (Gen)-loaded poly (lactic-co-glycolic acid) nanoparticles (GNPs) were coated with BsAb-MVs to form BsAb-functionalized GNPs (BsAb-GNPs). By leveraging high-affinity recognition of bacterial surface antigens and enhanced antibiotic penetration, BsAb-GNPs showed robust bactericidal activity by precisely targeting planktonic bacteria and biofilms, imposing effective local drug concentration. Moreover, BsAb-MVs retained antibody-mediated adhesion neutralization and host-cell invasion blockade, thereby mitigating toxin-induced tissue damage. *In vitro* biofilm assays and a murine pneumonia model confirmed the precise targeting and potent antibacterial efficacy of BsAb-GNPs. Collectively, this antibody-antibiotic system integrated multifunctional antibody-antibiotic therapeutic strategy for biofilm-associated infections and offers immunological advantages, which may provide a scalable and translational strategy against *P. aeruginosa* biofilm infections.

## Graphical abstract

**Figure:**
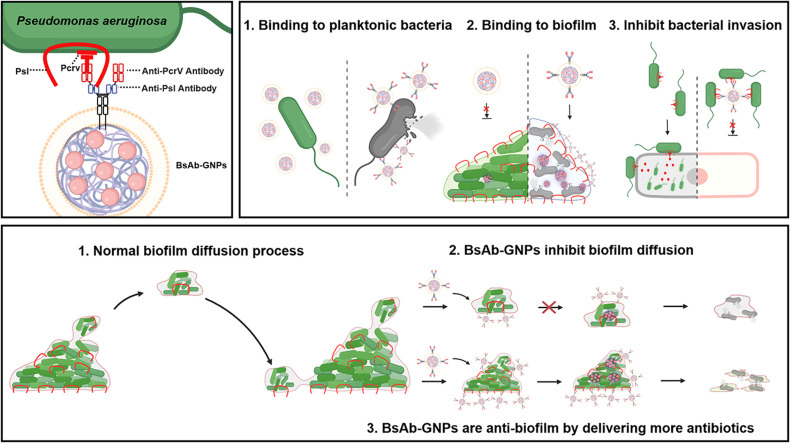

Schematic illustration of the BsAb-GNP mechanism of action in a murine model of *P. aeruginosa* pneumonia infection

## 1 Introduction

Bacterial infections pose a major threat to human health, with *Pseudomonas aeruginosa* (*P. aeruginosa*) being one of the most prevalent opportunistic pathogens responsible for pneumonia and wound infections [[1], [2], [3], [4], [5]]. It is widely distributed in diverse environments and causes high mortality, particularly among immunocompromised individuals, postoperative patients, and those in intensive care units (ICU) [6,7]. During acute infection, *P. aeruginosa* employs the type III secretion system (T3SS) to translocate virulence effectors into host cells via the PcrV needle-tip protein, thereby disturbing immune responses, impairing epithelial barriers and facilitating bacterial dissemination and sepsis [8]. In addition, *P. aeruginosa* secretes Psl polysaccharides that mediate adhesion to host cells and promote biofilm formation, conferring strong resistance to antibiotics, disinfectants, and host defenses, ultimately resulting in persistent chronic infections [9]. These biofilms are structured by an extracellular polymeric substance (EPS) matrix composed of Psl polysaccharides, extracellular DNA (eDNA), proteins, lipids and bacterial cells [10]. The current therapeutic strategy for pulmonary biofilm-associated *P. aeruginosa* infections primarily relies on high-dose antibiotics [11,12]. However, conventional small-molecule antibiotics suffer from limited penetration into biofilm EPS, short half-lives, poor targeting, inability to neutralize bacterial toxins, and rapid development of resistance [13]. Nanocarriers have been explored to encapsulate antibiotics and enhance drug penetration through biofilms, yet systemic administration may cause off-target cytotoxicity owing to their lack of targeting specificity [14]. Thus, how to effectively suppress bacterial virulence and selectively deliver antibiotics to biofilm infection sites simultaneously remains a critical challenge.

Before the advent of antibiotics, serum antibodies were employed to neutralize bacterial toxins or capsular polysaccharides [15]. Although later replaced by antibiotics due to superior safety and broad-spectrum efficacy, the rise of multidrug resistance and the difficulty of developing next-generation antibiotics have renewed interest in pathogen-specific antibody-based therapies [16]. For instance, monoclonal antibody (mAb) targeting Psl have been shown to mediate opsonophagocytic killing independent of bacterial serotype and to inhibit adhesion of *P. aeruginosa* to epithelial cells [17]. Similarly, anti-PcrV mAb can block the translocation of multiple T3SS effector proteins, thus reducing virulence [[18], [19], [20]]. However, the heterogeneity of virulent *P. aeruginosa* strains limits the efficacy of single mAb [21]. Advances in molecular engineering have enabled the construction of bispecific antibody (BsAb) by fusing single-chain variable fragments (scFv) with antibody heavy chains (HCs) or light chains (LCs) [22]. BsAb targeting both Psl and PcrV (MEDI3902) show improved protective efficacy and broader coverage against *P. aeruginosa* virulent strains. Nevertheless, the phase II clinical trial of MEDI3902 failed to demonstrate a statistically significant reduction of *P. aeruginosa* pneumonia in ventilated ICU patients, suggesting that antibody-only approaches may be insufficient in clinical settings [20,23]. This highlights the necessity of combining anti-virulence antibodies with conventional antibiotics to achieve robust therapeutic efficacy. Antibody-drug conjugates represent one possible strategy for such combination therapy, yet chemical conjugation may impair antibody function or mask antibiotic binding sites [24,25]. Therefore, an unmet need remains to co-deliver antibodies and antibiotics in a targeted manner to achieve combination antibacterial effects.

Recently, bottom-up approaches using cell membrane-coated nanoparticles have shown distinct advantages in antibacterial applications [26]. Such bioinspired nanostructures inherit biological functionalities including immune evasion, pathogen targeting, toxin neutralization, prolonged circulation, and drug delivery, which can be achieved through simple membrane-coating techniques [27]. Moreover, genetic engineering allows programmable enhancement of these biomimetic properties [28]. For example, our previous studies showed that hyaluronidase engineered on membrane vesicles can be released in a tumor microenvironment responsive manner, thus degrading the extracellular matrix and enhancing sonosensitizer penetration in deep tumor regions [29]. We also demonstrated that membrane vesicles displaying high-affinity receptors for hepatitis B virus entry can act as decoys to neutralize viral particles, thereby blocking virus replication and transmission [30]. Furthermore, we harnessed antibodies anchored membrane vesicles against methicillin-resistant *Staphylococcus aureus* (MRSA). It was shown that the vesicles can efficiently target MRSA PBP2a, penetrate alveolar barriers and enhance antibiotic accumulation at infection sites, resulting in effective treatment of MRSA-induced pneumonia [31]. Building on these findings, we hypothesized that membrane vesicles displaying antibodies dual-targeting Psl and PcrV could integrate biomimetic features with antibody bioactivity, thereby enabling efficient antibiotic delivery and multifunctional antibacterial activity.

**Fig. 1:**
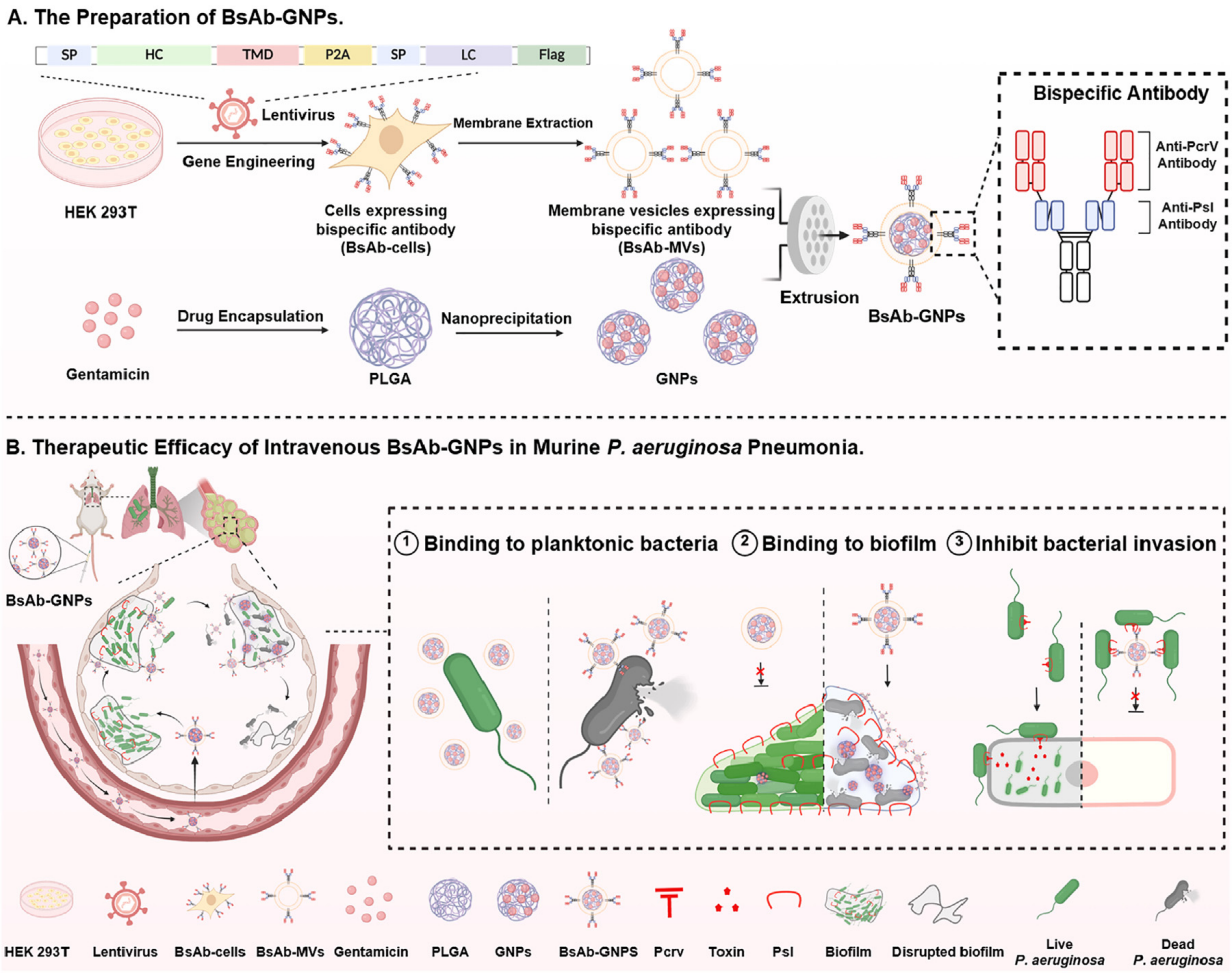
Preparation of BsAb-GNPs and their proposed mechanisms of action in a murine model of *P. aeruginosa* pneumonia infection. (A) Preparation of BsAb-GNPs. Cells stably expressing the BsAb (BsAb-cells) were generated via lentiviral transduction, and membrane vesicles were subsequently isolated to obtain BsAb-MVs. In parallel, Gen was encapsulated into PLGA using a nanoprecipitation method to form GNPs. BsAb-MVs were then coated onto the surface of GNPs to generate BsAb-GNPs. The lentivirally expressed fusion protein consists of a signal peptide (SP), BsAb (PcrV & Psl) light chain (LC) and heavy chain (HC), a transmembrane domain (TMD), and a flag tag; (B) Therapeutic efficacy of intravenous BsAb-GNPs in murine *P. aeruginosa* pneumonia. BsAb-GNPs enable targeted antibiotic delivery to both planktonic bacteria and biofilms via the BsAb, while simultaneously exerting antibody-mediated biological activity, thereby achieving combined antibiotic-antibody therapy.

Here, we presented a facile strategy that orchestrated multifunctional antibodies with nano-antibiotics to construct an antibody-functionalized nano delivery system for the combined treatment of *P. aeruginosa* infections. As a proof of concept, we firstly engineered a BsAb against *P. aeruginosa* by inserting an anti-Psl scFv into the hinge region, where the HC from an anti-PcrV mAb was settled as scaffold. The gene sequences of BsAb we designed were integrated into lentiviruses, and BsAb was subsequently expressed on the cell membrane surface. By physical extraction method, nanoscale membrane vesicles displaying BsAb (BsAb-MVs) were isolated. Concurrently, gentamicin (Gen) was encapsulated in poly (lactic-co-glycolic acid) (PLGA) nanoparticles (GNPs), which were then coated with BsAb-MVs to form BsAb-functionalized GNPs (BsAb-GNPs) (Fig. 1). This system leveraged the high-affinity binding of BsAb to *P. aeruginosa* surface antigens to achieve precise targeting of both planktonic cells and biofilms, thereby enhancing local antibiotic concentration within the infection microenvironment and improving penetration and bactericidal efficiency. Meanwhile, BsAb-MVs inherited antibody-mediated neutralization functions, suppressing bacterial adhesion and host-cell invasion, while mitigating toxin-induced tissue damage. Both *in vitro* biofilm models and *in vivo* murine pneumonia models demonstrated the superior targeting and antibacterial efficacy of BsAb-GNPs. Overall, this antibody-functionalized nano delivery platform enabled multifunctional therapy by combining antibiotics with antibody-mediated immunomodulation, thereby overcoming the limitations of traditional antibiotics in biofilm-associated infections and offering a novel, convenient and versatile strategy for combating drug-resistant *P. aeruginosa* infections.

## 2 Materials and methods

### 2.1 Materials

Gen, puromycin, PLGA, o‑phthalaldehyde (OPA), boric acid, glutaraldehyde, mercaptoethanol, DiO and Cy5.5 were purchased from Sigma (USA). Anti-Flag antibody and fluorophore-labeled anti-Flag primary were obtained from Abcam (UK). Human embryonic kidney cells (HEK293T) and human non-small cell lung carcinoma cells (A549) were maintained in this laboratory. HEK293T and A549 cells were cultured in Dulbecco’s Modified Eagle Medium (DMEM) supplemented with 10% fetal bovine serum (FBS) and 1% penicillin-streptomycin. *P. aeruginosa* PAO1, *P. aeruginosa* PAO1-GFP and *P. aeruginosa* PAO1-Lux strains were provided by Prof. Kangmin Duan and Prof. Lin Chen (Northwestern University). Bacteria were cultured in Luria-Bertani broth at 37 °C and enumerated on MacConkey agar plates.

### 2.2 Construction of BsAb-cells

Sequences of the BsAb were cloned to CMV-ZsGreen-Puro transfer plasmid (FENGHUI biotech, Hunan, China), and the sequencing result suggested successful construction of the recombinant plasmid. Lentiviruses carrying recombinant protein sequences were obtained by a three plasmids system. In brief, transfer plasmid (target protein), packaging plasmid (Gag), envelope plasmid (VSV-G) were added to 70%−80% confluency 293T cells by 4:3:2 (mass ratio) with lipofectamine 2000. The medium was refreshed at 16 h post‑transfection, and the supernatant was harvested at 48 h and 72 h, respectively. Lentiviruses were concentrated and purified by sucrose-gradient ultracentrifugation. The obtained lentiviruses were resuspended with PBS and stored at −80 °C prior to use.

HEK293T cells (5 × 10⁴ per well) were seeded in 24-well plates. At 60%−70% confluence, cells were infected with lentivirus carrying the BsAb fusion protein sequences in the presence of polybrene (5 µg/mL). After 24 h, the medium was replaced and cells were cultured for another 24 h. Transduction efficiency was monitored at 48 h by ZsGreen expression using confocal laser scanning microscopy (CLSM; Zeiss LSM900). At 72 h, puromycin (4 µg/ml) was added for 1 week to eliminate non-transduced cells. Surviving cells were expanded to establish a stable BsAb-expressing cell line.

### 2.3 BsAb-MVs extraction

When the cell growth reached 80%−90%, the culture medium was removed and the cells were rinsed once with PBS. Cells were gently scraped, transferred into PBS, and centrifuged at 2500 rpm for 5 min at 4 °C to remove residual medium. The pellet was resuspended in half the original volume of PBS and mixed with 5% sodium deoxycholate solution at a 100:1 (v/v) ratio. Cell disruption was performed in an ice-water bath using an ultrasonic processor at 15% power for 10–20 s. Subsequently, protease inhibitor (5 mg/ml) was added at a 200:1 (v/v) ratio. The mixture was centrifuged at 3500 rpm for 5 min at 4 °C to remove cytoplasmic and nuclear debris, followed by centrifugation at 15,000 rpm for 20 min at 4 °C to collect the BsAb-MVs pellet. Owing to the intrinsic fluidity of the phospholipid bilayer, the cell‑membrane fragments obtained after ultrasonic disruption and gradient centrifugation can spontaneously self‑assemble into nanoscale cell‑membrane vesicles. Depending on subsequent experimental requirements, the vesicles can then be extruded through 400‑nm polycarbonate membranes (Whatman) using a mini‑extruder (Avanti Polar Lipids) to produce cell‑membrane vesicles with uniform particle size. The obtained BsAb-MVs were resuspended in PBS by low-power sonication and stored at −80 °C until further use. The protein content of BsAb-MVs was determined using a BCA protein assay kit (Thermo Fisher, USA). Control MVs from cells without BsAb expression were prepared and quantified using the same procedure as mentioned above.

### 2.4 Characterization of BsAb-MVs

The characterization included immunofluorescence, flow cytometry and Western blotting. For immunofluorescence, cells were seeded in confocal culture dishes and incubated in an incubator overnight. After adhesion, cells were successively washed twice with PBS, fixed with 4% paraformaldehyde for 15 min, and washed once with PBS. Cells were then incubated with fluorophore-labeled anti-Flag primary antibodies for 1 h, followed by three PBS washes to remove unbound antibody. Nuclei were stained with DAPI for 10 min and washed three more times with PBS. Images were acquired using CLSM, with cells not expressing the BsAb serving as the control (Control-cells). For flow cytometry, cells were harvested, digested and washed, and the cell numbers in the experimental and control groups were adjusted to be equal. Cells were then subjected to fixation with 4% paraformaldehyde, PBS washing, incubation with Flag-labeled primary antibody, and additional PBS washes. Data were collected using a flow cytometer and analyzed with FlowJo software.

For Western blotting, cells were cultured in 10 cm dishes until reaching 90% confluence, washed twice with PBS, and lysed on ice for 30 min in lysis buffer containing protease inhibitors. Then the lysates were sonicated and centrifuged at 12,000 rpm for 30 min at 4 °C to collect supernatants. Protein concentrations were determined using the BCA assay, followed by mixing with loading buffer, boiling for 10 min, separation by SDS-PAGE, and transferred onto PVDF membranes. The membranes were blocked with 5% non-fat milk and sequentially incubated with Flag primary antibody and corresponding HRP-labeled secondary antibody, with PBS washes between steps to remove unbound antibodies. Protein bands were visualized and imaged. Western blotting of MV samples was performed using the same procedure after vesicle extraction.

### 2.5 Preparation of GNPs and BsAb-GNPs

GNPs were prepared by nanoprecipitation. Gen was dissolved in 0.5 ml of 1% polyvinyl alcohol (PVA) containing 0.95% MES buffer (pH 7) and added dropwise through a 25 G needle into 2 ml dichloromethane with 20 mg PLGA, under ultrasonication (80% power, 3 s on/3 s off, 20 cycles) and stirring at 1000 rpm, to form a primary water-in-oil emulsion. Next, 2.5 ml of this emulsion was slowly added to 10 ml PVA solution, followed by ultrasonication (80% power, 3 s on/3 s off, 60 cycles) and stirring at 1000 rpm on ice. The mixture was stirred for 4 h to remove dichloromethane, then washed twice by centrifugation and resuspension in PBS. The resulting GNPs were resuspended in cryoprotectant, freeze-dried and stored. Blank and fluorescent PLGA nanoparticles were prepared similarly, with light protection for the latter. BsAb‑GNPs were primarily prepared using a combined sonication and membrane‑extrusion method. Briefly, cell‑membrane vesicles were uniformly mixed with nanoparticles at a mass ratio of 1:2, followed by water‑bath sonication for 3 min. The mixture was then sequentially extruded through 400‑nm and 200‑nm polycarbonate membranes (Whatman) using a mini‑extruder (Avanti Polar Lipids), and subsequently purified by centrifugation. All other types of cell‑membrane‑coated nanoparticles were fabricated using the same procedure.

Gen content was quantified by the OPA method, in which OPA reacts with amino groups in Gen under alkaline conditions to form fluorescent isoindole derivatives. The reaction solution was prepared by dissolving 1 ml of 80 mg/ml OPA in ethanol, then adding 0.2 ml 0.4 M boric acid (pH 9.7), 0.2 ml ether and 0.4 ml mercaptoethanol. For measurement, 50 µl reaction solution was mixed with 50 µl Gen standard or sample in a black 96‑well plate, and fluorescence was recorded (Ex = 360 nm, Em = 460 nm). Gen content was calculated from a standard curve, and encapsulation efficiency (EE) and drug loading (DL) were determined accordingly. For release studies, GNPs or BsAb-GNPs stock solution (50 mg/ml, 200 µl) was diluted with PBS (pH 7.4 or 5.0, 0.8 ml) to a final concentration of 5 mg/ml and incubated at 37 °C with shaking (100 rpm). At predetermined intervals, the suspension was centrifuged (6765 × g, 5 min). Supernatant (50 µl) was collected for Gen quantification by the OPA method, and the pellet was resuspended in fresh PBS (50 µl) and returned to incubation. Gen concentration in the supernatant was determined using a standard curve. The cumulative release percentage, corrected for sampling dilution, was calculated relative to the total drug loading.

### 2.6 In vitro adhesion of BsAb-MVs to planktonic *P. aeruginosa*

Cy5.5-labeled BsAb-MVs were prepared by incubating BsAb-MVs with Cy5.5 for 30 min, followed by centrifugation and three times washes with cold PBS to remove unbound dyes. The labeled BsAb-MVs were then co-incubated with DAPI-stained *P. aeruginosa* PAO1 in PBS for 1 h. After centrifugation and three times washes, the bacterial pellets were resuspended, homogenized by pipetting, and filtered before analysis by using a flow cytometer (Beckman Cytoflex LX). Data were processed with FlowJo software, with MVs and untreated *P. aeruginosa* PAO1 serving as the control and blank groups, respectively. In parallel experiments, treated bacterial cells were mounted on slides and imaged by CLSM.

### 2.7 In vitro adhesion of BsAb-NPs to planktonic *P. aeruginosa*

Fluorescently dual-labeled BsAb-NPs were prepared by staining BsAb-MVs with DiO and NPs with Cy5.5. The labeled BsAb-NPs were co-incubated with DAPI-stained *P. aeruginosa* in PBS for 1 h, followed by centrifugation and three times washes. Bacterial adhesion to BsAb-NPs was quantified using a flow cytometer (Beckman Cytoflex LX), with data analyzed by FlowJo software. Control groups included NPs alone, MV-NPs, and untreated *P. aeruginosa* (blank control). In parallel experiments, processed samples were dried, sputter-coated with gold, and imaged by field-emission scanning electron microscopy (Gemini SEM 500). The same protocol was applied to MRSA for comparative analysis.

### 2.8 In vitro adhesion of BsAb-NPs to *P. aeruginosa* biofilms

A single colony of *P. aeruginosa* was inoculated into LB broth and cultured overnight at 37 °C. The overnight culture was then diluted 1:100 in fresh LB medium and activated for 4 h. After centrifugation, the bacterial suspension was adjusted to 1 × 10⁷ CFU/ml and added to 24-well plates containing polylysine-treated sterile cell climbing slides to establish biofilms during 24 h of static incubation. The culture medium was removed, and the biofilms were gently washed once with PBS, followed by DAPI staining. Fluorescently labeled BsAb-NPs were then incubated with the biofilms for 1 h. Unbound materials were washed away, and the climbing slides were imaged using a high-sensitivity confocal laser scanning microscope (Zeiss LSM 900) for layer-by-layer scanning. The three-dimensional (3D) structure of the biofilms was reconstructed using ZEN blue 3D software.

### 2.9 Animal care

All animal experiments were conducted in accordance with the Xiamen University Guidelines for the Care and Use of Laboratory Animals and approved by the Animal Ethics Committee of Xiamen University (XMULAC20190146). Eight-week-old male BALB/c mice were purchased from the Xiamen University Animal Center and housed under controlled conditions (22 °C, 60% humidity, and a 12 h light/dark cycle).

### 2.10 Targeting ability of BsAb-MVs in a mouse pneumonia model

A pneumonia model was established in mice via intratracheal instillation. Briefly, mice were fixed on a 60° inclined board, the tongue was gently pulled out, and a light-guided catheter with an indwelling needle was inserted through the oral cavity into the glottis. After confirming successful intubation, 50 µL of *P. aeruginosa* PAO1 suspension (∼5 × 10^6^ CFU in PBS) was instilled. Cy5.5-labeled BsAb-MVs were administered intravenously via the tail vein 2 h post-infection. After 24 h, major organs were collected for fluorescence imaging using an *in vivo* imaging system (Caliper IVIS Lumina), with MVs serving as the control.

### 2.11 BsAb biofunctional activity of BsAb-MVs in a mouse subcutaneous abscess model

A subcutaneous abscess model was established by injecting 100 µl of *P. aeruginosa* PAO1-Lux suspension (∼1 × 10^7^ CFU in PBS) into the subcutaneous tissue of the right hind leg. After 24 h, Cy5.5-labeled BsAb-MVs were injected intravenously via the tail vein. Bioluminescence and fluorescence imaging were performed at different time points using an *in vivo* imaging system (Caliper IVIS Lumina).

### 2.12 In vitro antibacterial activity of BsAb-GNPs against planktonic *P. aeruginosa*

The antibacterial activity of BsAb-GNPs against planktonic *P. aeruginosa* was evaluated by determining the minimum inhibitory concentration (MIC) and minimum bactericidal concentration (MBC). Free Gen, GNPs, MV-GNPs and BsAb-GNPs (Gen concentration 0–16 µg/ml, 100 µl) were mixed with *P. aeruginosa* PAO1 suspension in LB (100 µl, 2 × 10^5^ CFU/mL). Wells containing only materials without bacteria served as blank controls. After 24 h incubation, bacterial growth was assessed by measuring OD600. The MIC was defined as the lowest concentration with no visible bacterial growth. To determine the MBC, suspensions at the MIC and higher concentrations were plated on MacConkey agar and incubated at 37 °C for 24 h. The MBC was defined as the lowest concentration showing no colony formation.

### 2.13 In vitro anti-biofilm activity of BsAb-GNPs against *P. aeruginosa*

Biofilms were established by statically incubating *P. aeruginosa* PAO1-GFP (1 × 10^7^ CFU/ml) on poly-L-lysine-coated coverslips in 24-well plates for 24 h. For CLSM analysis, biofilms were washed with PBS, treated with Free Gen, GNPs, MV-GNPs, or BsAb-GNPs (Gen concentration 16 µg/ml) for 1 h, and further incubated for 4 h. Samples were washed, fixed with 4% paraformaldehyde for 30 min, and imaged by CLSM. Three-dimensional reconstructions and fluorescence intensity analyses were performed using Image J, and representative pseudocolor images were generated 7 µm above the biofilm base. PBS treated biofilms served as controls. For crystal violet staining, biofilms were washed, fixed with methanol for 15 min, air-dried, and stained with 0.01% crystal violet for 30 min. Excess dyes were removed by PBS washing, and bound crystal violet was solubilized with ethanol. Absorbance was measured to quantify residual biofilm biomass.

### 2.14 In vitro inhibition of *P. aeruginosa* invasion of epithelial cells by BsAb-NPs

BsAb-NPs were incubated with *P. aeruginosa* PAO1-GFP in PBS for 1 h and subsequently added to DAPI-stained A549 cells at a multiplicity of infection of 10:1. After 2 h, non-adherent bacteria were removed by PBS washing, and bacterial invasion was visualized by CLSM. In a parallel experiment, BsAb‑NPs were co‑incubated with *P. aeruginosa* PAO1 in PBS for 1 h and then added to A549 cells at an infection ratio of 10:1. After 2 h, non‑adherent bacteria were removed by PBS washing, and the cells were stained with calcein acetoxymethyl ester/propidium iodide (Calcein AM/PI) and examined under a fluorescence microscope. The uninfected cells were used as the control group and were denoted as the Sham group.

### 2.15 Protective effect of BsAb-GNPs in a murine pneumonia model

A pneumonia model was established as described above. Two hours post-infection, mice were treated with the indicated materials by tail vein injection (Gen dose: 2.5 mg/kg). After 24 h, lungs were collected, fixed with paraformaldehyde, and subjected to histological examination and Gram staining. For bacterial burden analysis, lungs were homogenized in pre-cooled PBS, and bacterial counts were determined by plate counting. For cytokine quantification, homogenates were centrifuged, and supernatants were analyzed using ELISA kits.

### 2.16 Cytotoxicity assay

Cytotoxicity was evaluated using the Cell Counting Kit-8 (CCK-8). Cells were seeded in 96-well plates at 5000 cells per well and incubated overnight at 37 °C with 5% CO₂. Cells were then treated with various concentrations of materials for 24 h. After removing the supernatant, 100 µL fresh medium and 10 µL CCK-8 reagent were added to each well, followed by incubation for 1 h. Absorbance was measured at 450 nm to assess cell viability. Untreated cells were defined as 100% viable.

### 2.17 In vivo safety evaluation

Mice received daily tail vein injections of materials (Gen dose: 2.5 mg/kg) for 3 consecutive days. On Day 14, major organs were collected and subjected to H&E staining for histopathological assessment of tissue toxicity.

### 2.18 Data analysis

All data are presented as mean ± SD from at least three independent experiments. Statistical analysis was performed using Student’s *t*-test for two-group comparisons and one-way or two-way ANOVA followed by Tukey’s post hoc test for multiple comparisons. Statistical significance was defined as *P < 0.05, ^⁎⁎^*P* < 0.01, ^⁎⁎⁎^*P* < 0.001 or ^⁎⁎⁎⁎^*P* < 0.0001. UD (undetectable) indicates values below the detection limit.

## 3 Results and discussion

### 3.1 Preparation and characterization of BsAb-GNPs

**Fig. 2:**
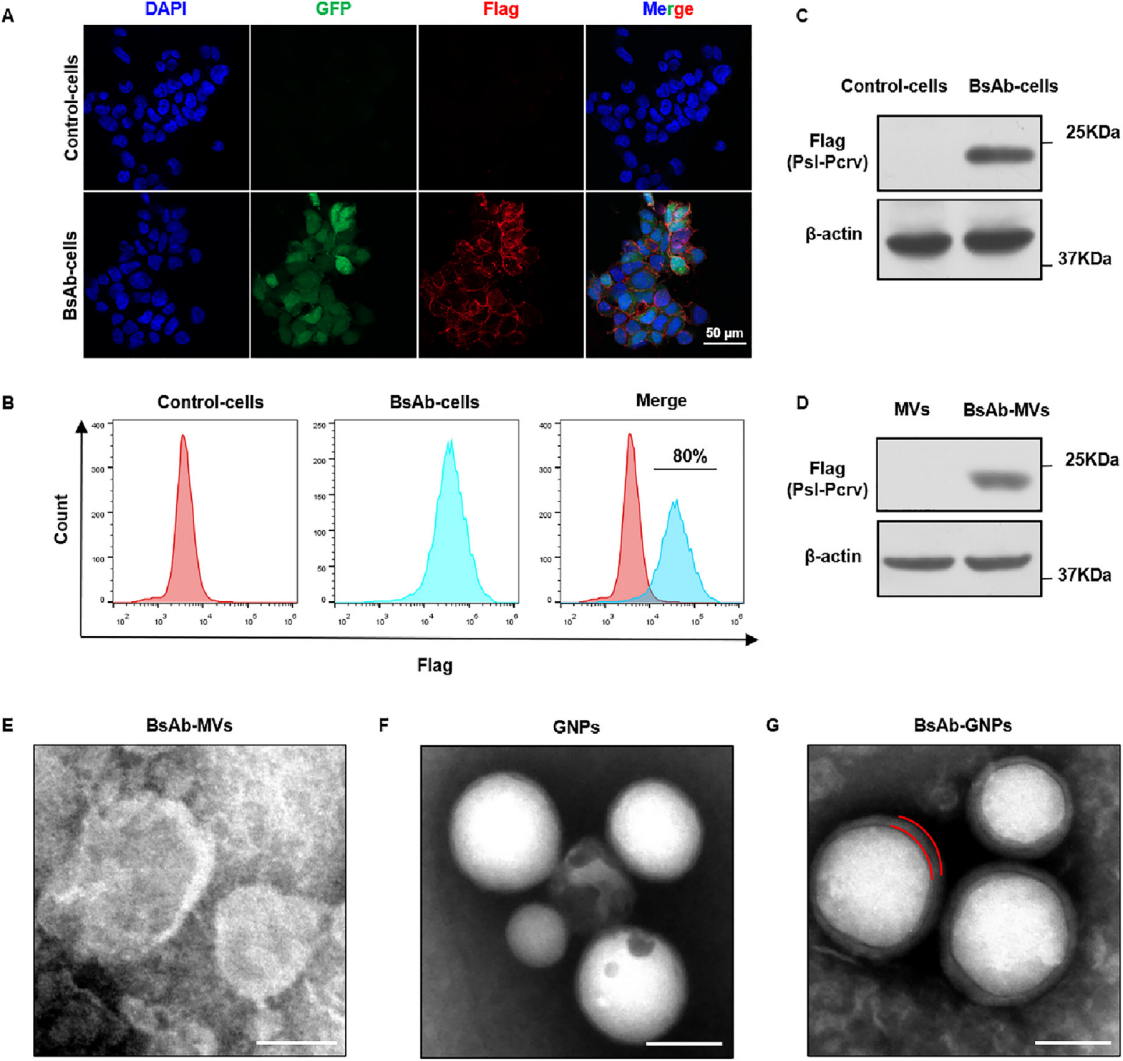
Characterization of BsAb-GNPs. (A) Immunofluorescence imaging of fusion protein expression in HEK293T cells under confocal microscopy. Scale bar: 50 µm. Blue: nuclei (DAPI); red: Flag tag; green: ZsGreen; (B) Flow cytometry analysis of fusion protein expression on the cell surface; (C) Western blot detection of fusion protein expression in transfected cells. (D) Western blot detection of fusion protein expression in BsAb-MVs; TEM image of (E) BsAb-MVs, and (F) GNPs. Scale bar: 100 nm; (G) TEM image of BsAb-GNPs showing core-shell structure, the red line indicates the cell membrane vesicle layer. Scale bar: 100 nm.

To display BsAb on the outer cellular membrane, we designed a fusion protein capable of anchoring to the plasma membranes. The fusion protein consists of SP, BsAb (PcrV&Psl) LC and HC, TMD, and a Flag tag. HEK293T cells were transduced with lentiviruses encoding this recombinant protein (Fig. S1). Following genomic integration, transcription and translation, the target protein was expressed and anchored on the cell surface. After puromycin selection, a stable cell line expressing BsAb was established. To verify the expression of this fusion protein on cellular membranes, flag protein was stained by fluorophore-labeled anti-Flag primary antibodies as an indicator. Under confocal microscopy, immunofluorescence imaging revealed that the red fluorescence signal corresponding to the Flag-tagged fusion protein was successfully detected on the cell membranes, while green fluorescence from the lentiviral vector (ZsGreen) was localized mainly in the cytoplasm (Fig. 2A). No fluorescence was detected in non-transduced control cells (Fig. 2A). By flow cytometry, it was confirmed that ∼80% of transfected cells expressed the fusion protein (Fig. 2B), and Western blotting further validated the BsAb expression by using anti-Flag antibodies (Fig. 2C). BsAb-MVs were subsequently extracted. Transmission electron microscope (TEM) analysis showed that BsAb-MVs exhibited typical vesicular morphology (Fig. 2E), and Western blot confirmed the presence of the fusion protein (Fig. 2D). Together, these results demonstrate the successful construction of a genetically engineered cell line expressing BsAb and the preparation of membrane vesicles displaying the BsAb.

Gen, a broad-spectrum first-line antibiotic, suffers from poor targeting, limited biofilm penetration, short half-life and intrinsic toxicity. These limitations restrict its clinical use, and nanotechnology may offer a means to optimize its therapeutic performance [32]. Here, we chose Gen as a model antibiotic and encapsulated it into PLGA nanoparticles via nanoprecipitation to prepare GNPs. The standard curve of Gen was established using the OPA method (Fig. S2), from which the DL and EE were calculated to be 2.5% and 10%, respectively. BsAb-MVs were subsequently coated onto the GNPs by membrane extrusion to generate BsAb-GNPs. TEM imaging showed that bare GNPs exhibited solid spherical morphology (Fig. 2F), whereas BsAb-GNPs displayed a clear core-shell structure with a distinct cell membrane vesicular coating around the GNP core (red outline) (Fig. 2G). The decreased zeta potential of GNPs after membrane coating indicates successful membrane wrapping (Fig. S3). The hydrodynamic diameter of BsAb-GNPs slightly increased due to membrane coating (Fig. S4). The polydispersity index (PDI) of BsAb-GNPs in PBS was 0.109, significantly lower than that of BsAb-MVs (0.557) or GNPs (0.368), indicating improved colloidal stability. This enhanced stability likely results from the GNP core supporting the fluid phospholipid bilayer of BsAb-MVs [33]. BsAb‑GNPs maintained a stable size and low PDI in serum, indicating strong potential for *in vivo* use (Fig. S5). Moreover, membrane coating conferred biomimetic camouflage, improved biocompatibility, reduced immune clearance, and prolonged circulation time, thereby creating a therapeutic window for targeted delivery [31]. Because bacterial infection sites typically exhibit an acidic microenvironment [34,35], BsAb‑GNPs showed faster drug release under acidic conditions than under neutral conditions (Fig. S6). This pH‑responsive behavior suggests that BsAb‑GNPs can preferentially release Gen at infection sites, reducing off‑target exposure and systemic toxicity. The accelerated release may result from the rapid degradation of the low‑molecular‑weight PLGA used (lactic acid:glycolic acid = 1:1) [32], as well as increased lipid fluidity and reduced stability of the membrane coating under acidic conditions.

### 3.2 In vitro biological functions mediated by BsAb-MVs

**Fig. 3:**
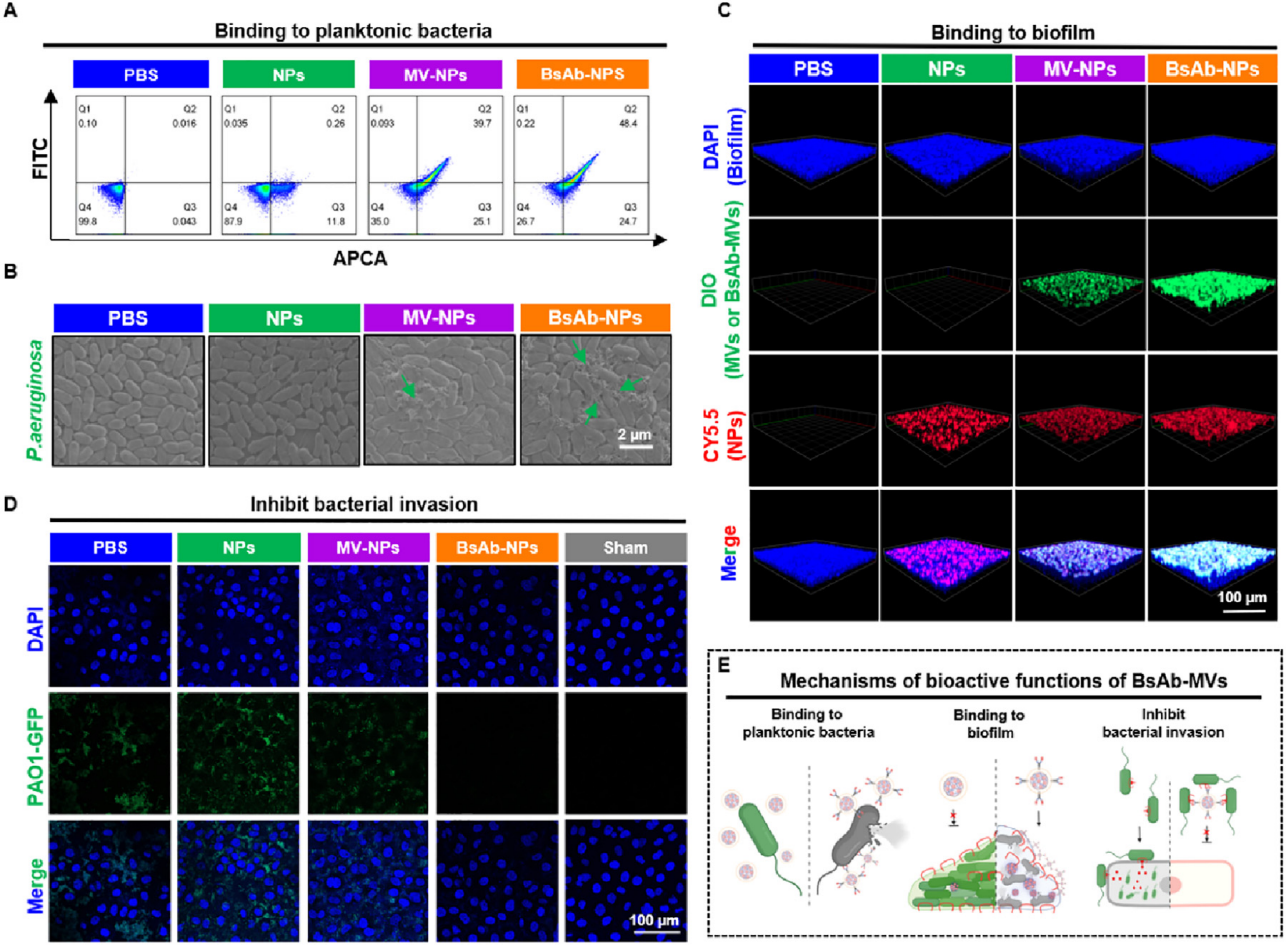
*In vitro* biological functions mediated by BsAb-MVs. (A) Flow cytometry analysis of BsAb-NP adhesion to planktonic *P. aeruginosa*; (B) SEM images showing adhesion of BsAb-NPs to planktonic *P. aeruginosa*. Green arrows indicate adhered nanoparticles. Scale bar: 2 µm; (C) CLSM images of nanoparticle delivery to mature *P. aeruginosa* biofilms by BsAb-MVs. Scale bar: 100 µm; (D) CLSM images of BsAb-NPs inhibiting *P. aeruginosa* PAO1-GFP invasion of lung epithelial cells. Scale bar: 100 µm, the Sham group represents uninfected cells; (E) Schematic illustration of BsAb-MVs mediating adhesion to planktonic *P. aeruginosa*, targeting biofilms, and blocking bacterial invasion.

Whereas BsAb against PcrV (a T3SS needle-tip protein on *P. aeruginosa*) and Psl (a biofilm EPS polysaccharide) were incorporated onto membrane vesicles, we hypothesized that high-affinity antigen-antibody interactions would enable selective adhesion of BsAb-MVs to both planktonic bacteria and biofilms. To assess adhesion to planktonic *P. aeruginosa*, BsAb-MVs and MVs were labeled with Cy5.5 and then co-incubated with bacteria. Flow cytometry revealed a ∼14% increase in adhesion for BsAb-MVs compared with MVs (Fig. S7), and CLSM confirmed enhanced attachment of BsAb-MVs to planktonic cells (Fig. S8). We next evaluated targeted nanodrug delivery ability of BsAb-MVs to planktonic bacteria using dual-fluorescent materials, including DiO-labeled vesicles and Cy5.5-labeled PLGA nanoparticles (NPs). After co-incubation with *P. aeruginosa*, flow cytometry revealed that BsAb-NPs exhibited higher binding than bare NPs and MV-NPs (Fig. 3A and S9). Scanning electron microscopy (SEM) confirmed denser nanoparticle adhesion on bacterial surfaces in the BsAb-NP group, whereas fewer NPs or MV-NPs were observed (Fig. 3B). Notably, BsAb-NPs did not adhere to MRSA, indicating selectivity for *P. aeruginosa* (Fig. S10). These results demonstrate that antibody functionalization enhances MV adhesion to planktonic *P. aeruginosa* and improves targeted nanodrug delivery.

Since *P. aeruginosa* readily forms biofilms, and its EPS not only hinders the contact between antibiotics and bacteria and weakens the antibacterial effect, but also envelops the target molecules on the *P. aeruginosa* cell surface, thus limiting the design of targeted drugs. Although nanosized antibiotics may possess enhanced penetration into biofilms than small molecule antibiotics, their therapeutic efficacy *in vivo* is still limited due to the lack of targeting. Given the abundance and structural role of Psl across biofilm maturation, we tested whether BsAb-MVs could adhere to and deliver cargo within mature biofilms. Dual-fluorescent materials as mentioned above were co-incubated with established biofilms. CLSM showed markedly fewer red fluorescent in the NPs group than in the BsAb-NPs group (Fig. S11); both green vesicles and red NP signals were higher in BsAb-NPs than in MV-NPs (Fig. 3C). This suggests that BsAb-MVs enhance the retention and penetration of GNPs in biofilms, thereby potentially improving therapeutic effects.

A critical step for bacterial infection is the adhesion of pathogens to host cells, a process mediated by multiple virulence factors. *P. aeruginosa* adheres to host cells via the Psl polysaccharide and employs the needle tip protein PcrV of the T3SS to inject toxins into cells, thereby disrupting membrane integrity and promoting bacterial internalization [23]. Blocking this invasion step could effectively prevent infection progression at an early stage. As demonstrated above, BsAb-MVs can efficiently adhere to the surface of planktonic *P. aeruginosa*. Given that *P. aeruginosa* is a predominant cause of pulmonary infection, we next evaluated the ability of BsAb-NPs to inhibit its invasion of lung epithelial cells. NPs, MV-NPs or BsAb-NPs were firstly co‑incubated with *P. aeruginosa* PAO1‑GFP, followed by infection of epithelial cells. CLSM imaging revealed that the NPs group exhibited green fluorescence intensity comparable to PBS, with signals surrounding nuclei, indicating intracellular bacteria and a lack of inhibitory effect. The MV‑NPs group showed moderately reduced fluorescence, possibly due to partial attenuation of bacterial invasion through interactions between pathogen and mammalian membranes. In contrast, the BsAb‑NPs group displayed the fewest invading bacteria on cell surfaces, suggesting that the combination of multivalent anti‑invasion antibodies and enhanced bacterial binding efficiency conferred optimal inhibition of *P. aeruginosa* invasion (Fig. 3D). Such inhibition of bacterial invasion also protects host cells from bacteria‑mediated cytotoxicity (Fig. S12). These findings indicate that genetically engineered membrane vesicles displaying BsAb can not only exploit high‑affinity antigen binding for targeted drug delivery, but also retain intrinsic antibody bioactivity to effectively block bacterial invasion by neutralizing key virulence factors (Fig. 3E). Compared with previous approaches that used pathogen‑derived membranes for anti‑invasion therapy, the strategy we proposed offers greater flexibility, specificity and safety.

### 3.3 In vitro antibacterial activity of BsAb-GNPs

**Fig. 4:**
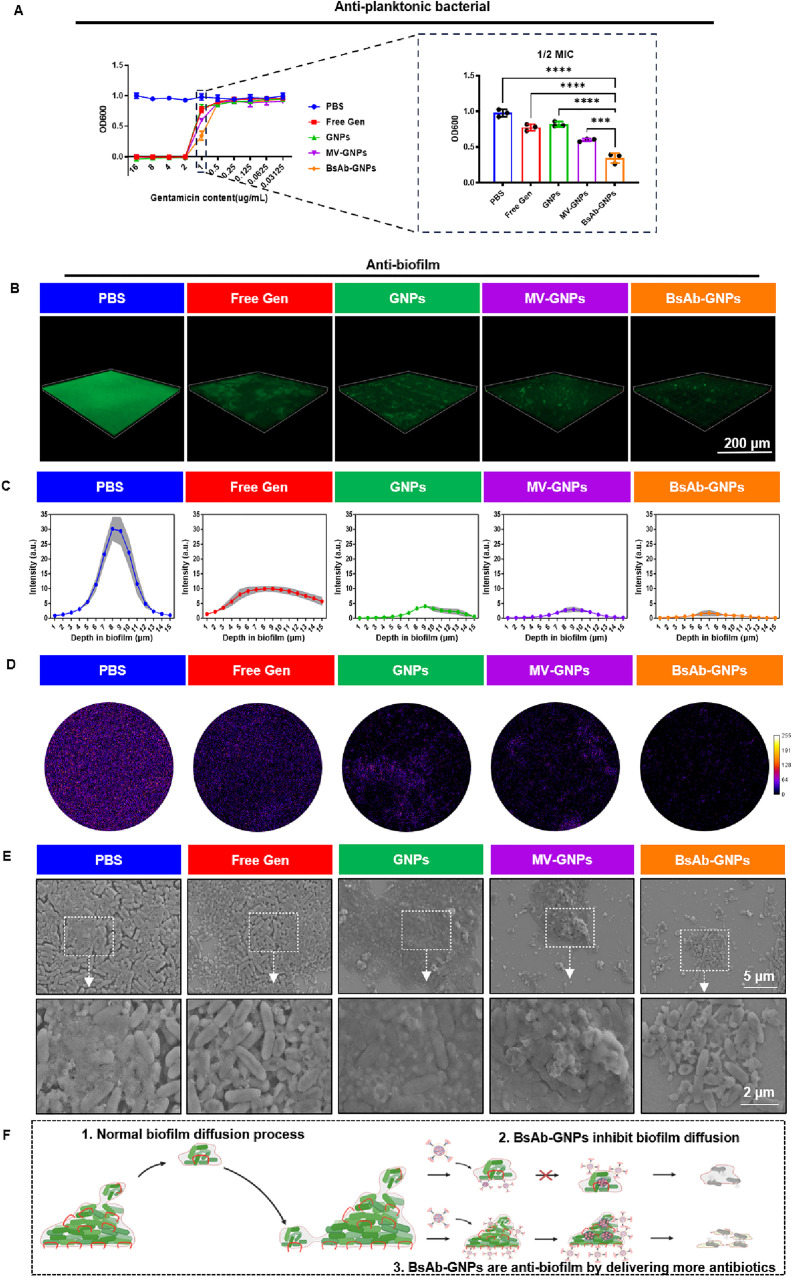
*In vitro* antibacterial activity of BsAb-GNPs. (A) Inhibitory effect of BsAb-GNPs against planktonic *P. aeruginosa*. The dashed line indicates inhibition at 1/2 MIC; (B) CLSM images of *P. aeruginosa* PAO1-GFP biofilms after treatment with different groups. Scale bar: 200 µm; (C) Representative pseudocolor CLSM images at a plane 7 µm above the biofilm base following treatment with different groups; (D) Quantitative fluorescence intensity profiles across biofilm depth obtained from CLSM images after treatment with different groups; (E) SEM images of *P. aeruginosa* biofilms following treatment with different groups, including magnified views. Scale bar: 5 µm and 2 µm for upper and lower panels, respectively; (F) Proposed mechanisms of BsAb-GNPs in antibiofilm activity: (1) normal biofilm expansion process; (2) BsAb-GNPs inhibit biofilm spread by blocking Psl polysaccharide adhesion; (3) BsAb-GNPs enhance antibiofilm efficacy by delivering higher concentrations of antibiotics within the biofilm. Statistical analysis was performed using one-way ANOVA followed by Tukey’s post hoc test for multiple comparisons. Statistical significance was defined as ^⁎⁎⁎^*P* < 0.001 or ^⁎⁎⁎⁎^*P* < 0.0001.

To assess the antibacterial efficacy of BsAb-GNPs, we firstly determined the MIC and MBC against planktonic *P. aeruginosa*. The MIC values of GNPs, MV-GNPs and BsAb-GNPs were identical to that of Free Gen (2 µg/ml), indicating that PLGA encapsulation and membrane coating did not compromise the intrinsic activity of Gen (Table S1). At half the MIC, BsAb-GNPs exhibited superior inhibitory activity compared with other groups, and their MBC values were also lower, suggesting that BsAb-GNPs not only delayed the growth of planktonic *P. aeruginosa* but also enhanced bactericidal efficacy (Fig. 4A).

Since biofilm formation is a major cause of drug resistance and recurrent chronic infections in *P. aeruginosa*, effective biofilm eradication is essential for effective therapy. Our previous results demonstrated that BsAb-MVs inhibited bacterial invasion and facilitated nanoparticle delivery into biofilms, raising the possibility that combining with nano-antibiotics could improve anti-biofilm outcomes. Based on this rationale, we next evaluated the antibiofilm activity of BsAb-GNPs. CLSM was used to perform z-stack scanning and reconstruct 3D images of biofilms treated with different groups. As shown in Fig. 4B, the control group displayed intact biofilm morphology, whereas BsAb-GNPs treatment resulted in the most severe structural disruption. Free Gen, GNPs and MV-GNPs also caused visible damage to biofilm integrity, but their effects were less pronounced compared with BsAb-GNPs. Representative pseudocolor CLSM images at 7 µm above the biofilm base further confirmed that BsAb-GNPs led to the lowest colony density and smallest number of bacterial clusters (Fig. 4C). Quantification of fluorescence intensity across different biofilm depths demonstrated that BsAb-GNPs consistently reduced fluorescence signals compared with other groups, indicating a more effective reduction of biofilm thickness (Fig. 4D).

To further investigate the mechanism underlying the antibiofilm activity, we examined biofilm surfaces treated with different groups using SEM. In the PBS group, bacteria were densely packed within the biofilm, covered by a thick extracellular matrix, forming a compact 3D network with smooth, intact bacterial membranes. In the Free Gen group, bacterial membranes showed wrinkles and ruptures, but overall biofilm structure remained intact, likely due to the limited penetration of Free Gen through the biofilm. The GNPs and MV-GNPs groups displayed partial structural disruption, yet clusters of intact biofilm aggregates persisted, suggesting that while GNPs improved adhesion and penetration compared with free drug, they were insufficient for complete eradication, potentially allowing recurrent infection. In contrast, BsAb-GNPs caused extensive destruction of the biofilm architecture. Residual bacteria adhering to silicon substrates exhibited perforated membranes with cytoplasmic leakage, confirming that BsAb-GNPs effectively disrupted both biofilm structure and bacterial cell membranes, thereby ensuring complete bacterial killing (Fig. 4E). Finally, we quantified residual biofilm biomass by crystal violet staining. Among all groups, BsAb-GNPs treatment resulted in the lowest biomass and the highest biofilm clearance rate, further validating their superior antibiofilm capability (Figs. S13 and S14). These results suggest that BsAb-GNPs enhance biofilm eradication and it may be attributed to two mechanisms: (1) antibody-mediated adhesion enables higher accumulation and deeper penetration of Gen, and (2) anti-Psl antibodies prevent new biofilm formation by residual planktonic bacteria (Fig. 4F).

### 3.4 In vivo biological functions mediated by BsAb-MVs

**Fig. 5:**
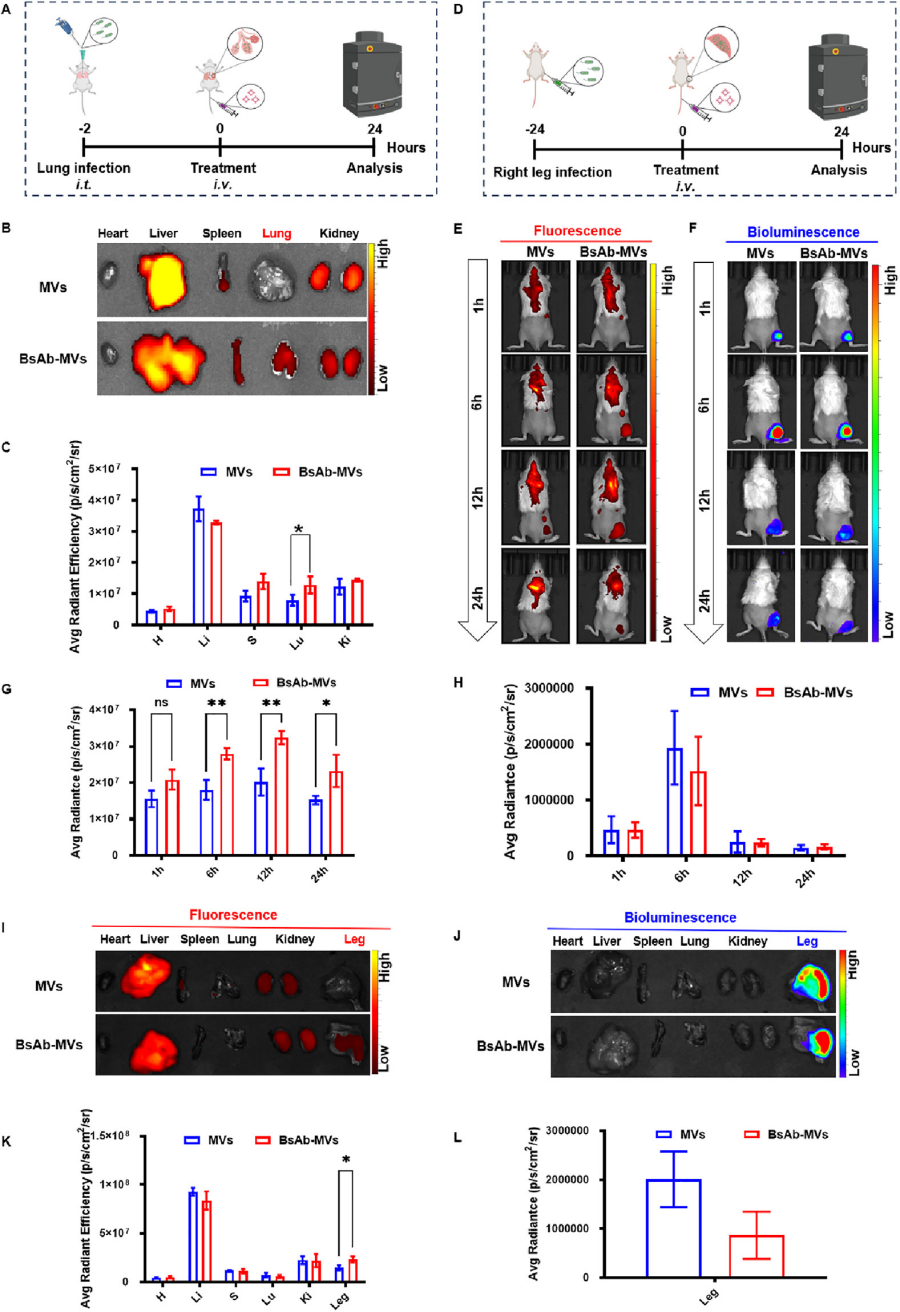
*In vivo* biological functions mediated by BsAb-MVs. (A) Schematic of pneumonia model establishment and administration; (B) *Ex vivo* fluorescence imaging of organs from pneumonia mice; (C) Quantitative *ex vivo* fluorescence analysis (*n* = 3). (D) Schematic of subcutaneous abscess model and administration; (E) *In vivo* fluorescence images of subcutaneous abscess mice; (F) *In vivo* bioluminescence images of subcutaneous abscess mice; (G) Quantification of *in vivo* fluorescence (*n* = 3); (H) Quantification of *in vivo* bioluminescence (*n* = 3); (I) *Ex vivo* fluorescence images of organs and infected leg; (J) *Ex vivo* bioluminescence images of organs and infected leg; (K) Quantification of *ex vivo* fluorescence of organs and infected leg (*n* = 3); (L) Quantification of *ex vivo* bioluminescence of infected leg (*n* = 3). Statistical analysis was performed using two-way ANOVA followed by Tukey’s post hoc test for multiple comparisons. Statistical significance was defined as *P < 0.05, **P < 0.01.

*P. aeruginosa* is one of the principal causative agents of bacterial pneumonia. The presence of airway mucus and *P. aeruginosa* biofilms in the lung severely hinders targeted delivery of antibacterial drugs to infected sites. *In vitro* studies showed that BsAb-MVs adhered more efficiently to *P. aeruginosa* than unmodified vesicles, suggesting the potential for systemic circulation and preferential accumulation at infected lung tissue. To evaluate the *in vivo* targeting capability of BsAb-MVs toward pulmonary *P. aeruginosa*, a non-invasive mouse pneumonia model was established via intratracheal inoculation, followed by intravenous administration of Cy5.5-labeled MVs or BsAb-MVs (Fig. 5A). After 24 h post injection, *ex vivo* fluorescence imaging of harvested organs revealed that MVs generated only weak signals in the lungs, with predominant red fluorescence in the liver, indicating primary hepatic clearance after circulation (Fig. 5B). In contrast, BsAb-MVs exhibited reduced liver accumulation and approximately two-fold higher fluorescence intensity in infected lung tissue compared with MVs, indicating enhanced adhesion and retention at the infection site (Fig. 5C).

To further validate the adhesion of BsAb-MVs to *P. aeruginosa in vivo*, a murine subcutaneous abscess model was established using the bioluminescent strain *P. aeruginosa* PAO1-Lux (Fig. 5D). Bioluminescence imaging confirmed successful colonization of *P. aeruginosa* PAO1-Lux in the subcutaneous tissue of mouse hind limbs (Fig. 5F). Cy5.5-labeled vesicles were then administered intravenously, and both fluorescence and bioluminescence signals were captured at 1 h, 6 h, 12 h and 24 h post-injection. Mice were sacrificed at 24 h for *ex vivo* organ imaging. *In vivo* fluorescence imaging revealed that MVs mainly accumulated in the liver, with only minimal localization at the infection site at 12 h, and their signals nearly disappeared by 24 h. By contrast, BsAb-MVs were detected at the infection site as early as 6 h, colocalizing with *P. aeruginosa* PAO1-Lux, and exhibited prolonged retention at the infected tissue, highlighting their superior specificity and targeting ability towards *P. aeruginosa* (Fig. 5E and 5G). Consistently, *ex vivo* fluorescence signals in the BsAb-MV group were stronger than those in the MV group at 24 h, further confirming improved adhesion to infected tissues (Fig. 5I and 5K). Bioluminescence imaging demonstrated that at 6 h, the intensity of *P. aeruginosa* PAO1-Lux signal in the BsAb-MV group was reduced compared with the MVs group (Fig. 5F and 5H). At 24 h, the *ex vivo* bioluminescence signal of *P. aeruginosa* PAO1-Lux in infected tissues of the BsAb-MV group was approximately half that observed in the MV group (Fig. 5J and 5L). These findings indicate that BsAb-MVs can effectively inhibit the growth of *P. aeruginosa* PAO1-Lux *in vivo* and delay biofilm formation. This enhanced therapeutic effect may be attributed to the multiple anti-invasion antibody copies on BsAb-MVs, which facilitate bacterial recognition and growth inhibition, thereby providing a promising strategy for encapsulating nano-antibiotics within BsAb-MVs for targeted treatment of bacterial infections.

### 3.5 Protective effects of BsAb-GNPs in a mouse pneumonia model

**Fig. 6:**
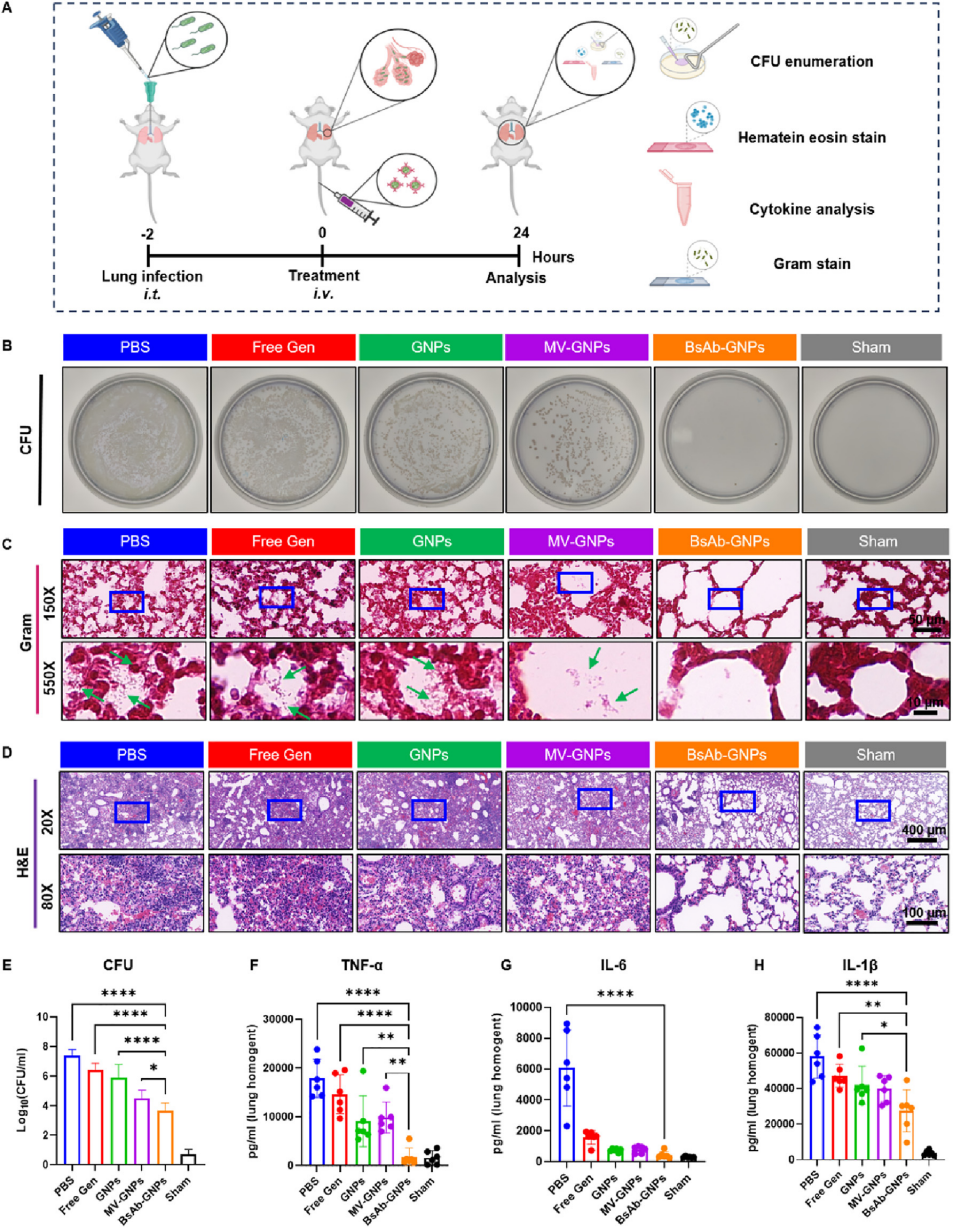
Protective effects of BsAb-GNPs in a murine pneumonia model. (A) Schematic of pneumonia model establishment and treatment; (B) Images of viable bacteria in lung homogenates; (C) Gram-stained lung sections, with Gram-negative bacterial colonies and individual cells indicated by green arrows in the magnified views, scale bars: 50 µm and 10 µm for upper and lower panels, respectively; (D) H&E-stained lung sections. scale bars: 400 µm and 100 µm for upper and lower panels, respectively; (E) Quantification of viable bacteria in lung homogenates (*n* = 6); (F-H) ELISA analysis of TNF-α, IL-6 and IL-1β levels in lung tissues (*n* = 6). Statistical analysis was performed using one-way ANOVA followed by Tukey’s post hoc test for multiple comparisons. Statistical significance was defined as *P < 0.05, **P < 0.01 or ****P < 0.0001.

Based on the inspiring targeting and antibacterial activity of BsAb-GNPs observed *in vitro*, we further evaluated their therapeutic efficacy in a mouse *P. aeruginosa* pneumonia model. As shown in Fig. 6A, mice were inoculated intratracheally with *P. aeruginosa*, and 2 h later, PBS, Free Gen, GNPs, MV-GNPs or BsAb-GNPs were intravenously administered. A Sham group was included to exclude the influence of the surgical procedure itself. To assess the *in vivo* antibacterial efficiency, bacterial loads in lung homogenates were determined (Fig. 6B). Compared with PBS treatment, BsAb-GNPs reduced the bacterial burden in the lungs by 3.66 log units (Table S2). Moreover, the bacterial counts in the BsAb-GNPs group were significantly lower than those in the MV-GNPs group, indicating that BsAb-GNPs exhibited the strongest antibacterial effect against *P. aeruginosa* pneumonia (Fig. 6E). Gram staining of lung sections was then performed to visualize bacterial distribution (Fig. 6C). In the PBS, Free Gen, GNPs and MV-GNPs groups, abundant Gram-negative bacterial colonies adhered to alveolar epithelial cells and aggregated into microcolonies, representing the formation and spread of biofilms (green arrows). In contrast, BsAb-GNPs treatment resulted in fewer bacteria, which appeared as scattered individuals. These findings suggest that BsAb-GNPs not only suppress biofilm formation but also achieve combined antibody-antibiotic therapy to eradicate bacteria embedded within biofilms, thereby potentially preventing recurrent infections caused by residual bacteria.

The virulence factors of *P. aeruginosa* may further aggravate infection. The secreted Psl polysaccharide serves as both a structural scaffold for biofilm stability and a pathogen-associated molecular pattern (PAMP) that activates neutrophils, which can lead to excessive immune responses. In addition, toxins secreted via the PcrV needle protein of the T3SS can cause acute inflammatory injury. Previous studies have shown that mAb against Psl or PcrV can mitigate the acute inflammatory responses associated with *P. aeruginosa* infection. To verify whether Psl and PcrV dual-targeting antibodies displayed on membrane vesicles retained their anti-inflammatory activity, we next investigated whether BsAb-GNPs could attenuate *P. aeruginosa*-induced lung injury and inflammation. Histological analysis of lung tissues was performed by H&E staining (Fig. 6D). In the Sham group, alveoli were intact with open airspaces and showed no inflammatory cell infiltration, confirming that model establishment did not harm lung tissues. In contrast, PBS-treated mice displayed alveolar fusion and dilation, which is indicative of severe infection. Free Gen, GNPs and MV-GNPs groups also failed to provide complete protection, with varying degrees of alveolar septal thickening, septal rupture, and intra-alveolar congestion and edema. Strikingly, BsAb-GNPs treatment resulted in only mild lung injury, with preserved alveolar structure, intact epithelial cells, and reduced inflammatory cell infiltration. To further evaluate the inflammatory response, representative cytokines including TNF-α, IL-6 and IL-1β were measured in lung homogenates by ELISA (Fig. 6F–6H). Compared with PBS, BsAb-GNPs significantly reduced the levels of the typical pro-inflammatory cytokines, indicating that BsAb-GNPs not only suppressed bacterial infection but also alleviated the associated inflammatory response.

The design of BsAb was originally intended to address the limited efficacy of single-target antibodies in the face of pathogen heterogeneity. Indeed, the phase II trial of MEDI3902 failed to significantly reduce *P. aeruginosa* infections in ICU patients, suggesting that antibody blockade alone may not meet the demands of complex clinical infections [36]. Our approach, which combines BsAb-functionalized vesicles with antibiotic nanoparticles, provides a dual-hit mechanism “anti-virulence blockade + bactericidal activity” that surpasses antibody therapy alone. Furthermore, unlike conventional nanocarriers such as liposomes or polymeric nanoparticles, which largely rely on passive accumulation and are often insufficient in biofilm environments [32], BsAb-MVs enable active recognition through antibody-mediated targeting. This “active targeting + passive diffusion” synergy enhances antibiotic localization and penetration, thereby improving bacterial clearance and biofilm eradication. Such a mechanism may offer a new avenue to address biofilm-associated antimicrobial resistance and to overcome the uneven pulmonary distribution and limited efficacy of inhaled antibiotics. From a broader perspective, the BsAb-targeted nano antibiotic delivery strategy could be extended to other multidrug-resistant pathogens and, when combined with inhaled administration, may provide a precise therapeutic option for lower respiratory tract infections.

### 3.6 Biosafety evaluation of BsAb-GNPs

**Fig. 7:**
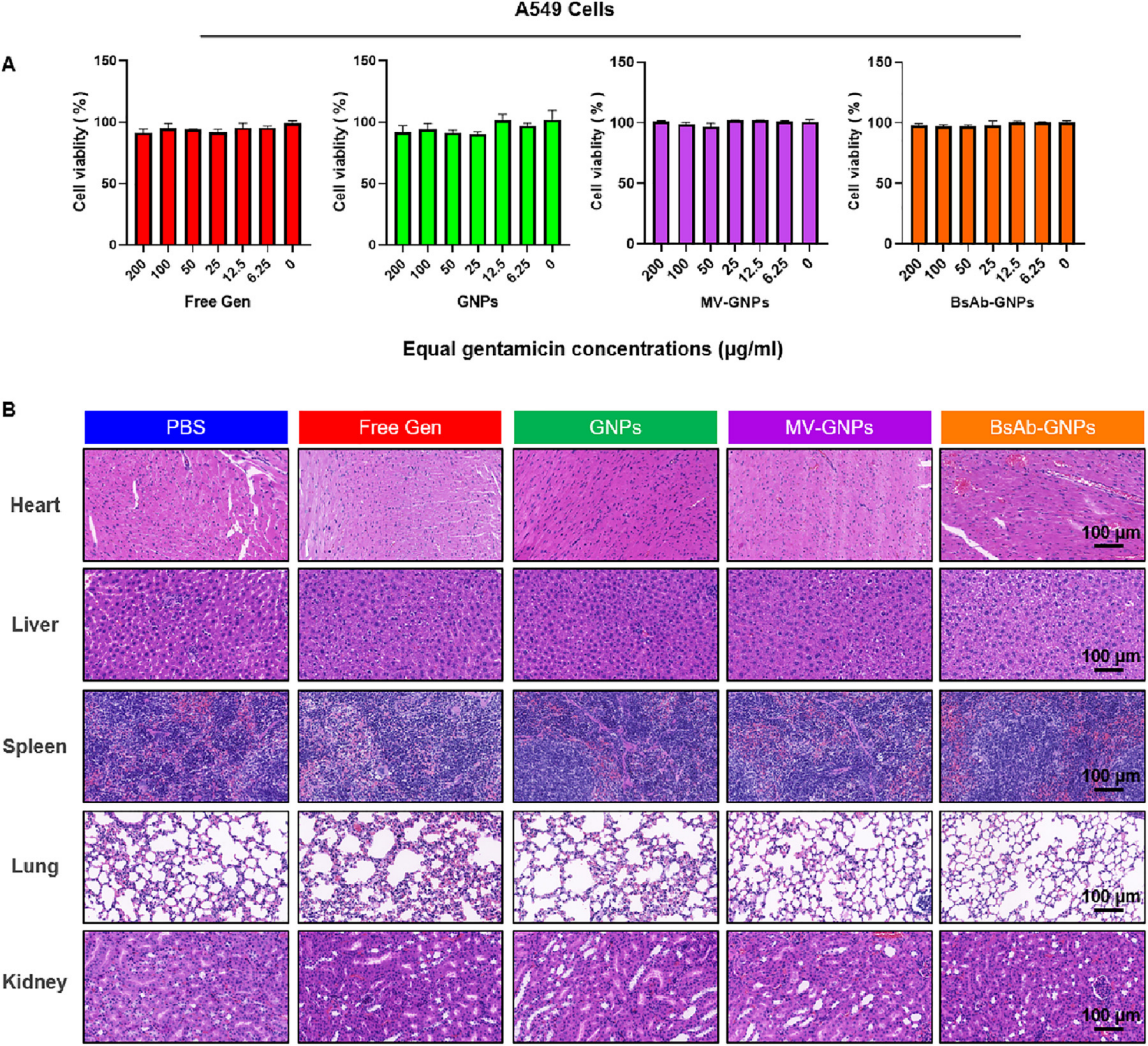
*In vitro* and *in vivo* biosafety evaluation of the drug. (A) Viability of A549 cells after treatment with the materials (*n* = 3); (B) H&E-stained sections of major organs from mice on Day 14 following intravenous administration of different materials, scale bar: 100 µm.

We evaluated the biosafety of the different treatments both *in vitro* and *in vivo*. Alveolar epithelial cells, which serve as the primary sites for *P. aeruginosa* colonization and invasion during bacterial pneumonia, were selected for experimental evaluation. The cytotoxicity of BsAb-GNPs was assessed using a standard CCK-8 assay. The data showed that Free Gen, GNPs, MV-GNPs and BsAb-GNPs all exhibited excellent biocompatibility, with cell viability exceeding 90% after 24 h of treatment at the tested concentrations (Fig. 7A). In addition, we assessed the potential long-term side effects of Free Gen, GNPs, MV-GNPs and BsAb-GNPs in healthy mice. During a 14-day observation period, no mortality was recorded, and histological analysis of major organs revealed no apparent pathological changes (Fig. 7B). The biosafety of BsAb-GNPs is attributed to their composition: cell membranes with inherent biocompatibility, PLGA as an FDA-approved polymer carrier, and Gen as a frontline antibiotic, all of which are considered relatively safe. Overall, these findings indicate that BsAb-GNPs are generally tolerant *in vivo* and demonstrate excellent biosafety, supporting their potential for therapeutic application against *P. aeruginosa* infections.

## 4 Conclusions

The biofilm and virulence factors of *P. aeruginosa* are known to cause pulmonary infections and significantly reduce the efficacy of conventional antibiotics and antibody therapies. In this study, a novel strategy was developed to combine multifunctional BsAb-MVs with nano-antibiotics, enabling targeted delivery of the nano-antibiotics while exerting multivalent anti-invasion activity, finally establishing an *in situ* combined therapeutic system characterized by “BsAb-targeted navigation + nano-antibiotic biofilm penetration + antibody-mediated interference”. Specifically, BsAb were displayed on cell membrane vesicles (BsAb-MVs) by genetic engineering, allowing specific binding to *P. aeruginosa* planktonic cells and biofilms *in vivo*, inhibiting bacterial adhesion to cells and tissues, and serving as carriers for precise delivery of nano-antibiotics to infection sites. The antibacterial efficacy of this system was confirmed in both *in vitro* biofilm assays and *in vivo* mouse pneumonia models, demonstrating enhanced antibacterial effects through the combination of antibodies and antibiotics. Overall, this study represents the innovative and subtle integration of *P. aeruginosa*-targeted BsAb-MVs with nano-antibiotics, providing a facile approach for the development of precisely targeted antibacterial nanomedicines. This study illustrates the potential of genetic engineering to extend cell membrane coating strategies, and offer a versatile platform potentially applicable to other bacterial infections.

## Conflicts of interest

The authors declare that there is no conflicts of interest.

## CRediT authorship contribution statement

**Jiaxin Ma:** Writing – review & editing, Visualization, Supervision, Methodology, Funding acquisition, Data curation, Conceptualization. **Zihao Teng:** Writing – original draft, Visualization, Software, Resources, Project administration. **Xuqi Peng:** Project administration, Methodology, Investigation. **Yanyin Wang:** Project administration. **Linyu Ding:** Project administration. **Yijia Xie:** Project administration. **Wenhui Huang:** Project administration. **Qiuyue Long:** Project administration. **Jianzhong Zhang:** Supervision. **Lai Jiang:** Writing – original draft, Supervision. **Gang Liu:** Writing – review & editing, Supervision, Funding acquisition, Conceptualization.

## Appendix Supplementary materials

Image, application 1
