# Supplementary material for: Bispecific antibody vesicles: A multifunctional bioactive drug delivery platform for the treatment of Pseudomonas aeruginosa infection
Source: Asian J Pharm Sci. 2026 May 17;21(3):101162. doi: 10.1016/j.ajps.2026.101162 (PMC13261987; doi:10.1016/j.ajps.2026.101162)
Supplement: Supplementary file 1 [file mmc1.docx]

**Supplementary Information**

**Bispecific antibody vesicles: a multifunctional bioactive drug delivery platform for the treatment of *Pseudomonas aeruginosa* infection**

Jiaxin Ma ^a #^, Zihao Teng ^b #^, Xuqi Peng ^a #^, Yanyin Wang ^a^, Linyu Ding ^a^, Yijia Xie ^a^, Wenhui Huang ^a^, Qiuyue Long ^c^, Jianzhong Zhang ^d^ *, Lai Jiang ^e^ *, Gang Liu ^a^ *

a State Key Laboratory of Vaccines for Infectious Diseases, Xiang An Biomedicine Laboratory, National Innovation Platform for Industry-Education Integration in Vaccine Research, Fujian Engineering Research Center of Molecular Theranostic Technology, Center for Molecular Imaging and Translational Medicine, School of Public Health, Xiamen University, Xiamen 361102, China

b School of Pharmaceutical Sciences, Xiamen University, Xiamen 361102, China

c School of Medicine, Xiamen University, Xiamen 361102, China

d Department of Neurological Surgery, Feinberg School of Medicine, Northwestern University, Chicago, Illinois, USA.

e School of Pharmaceutical Sciences, Zhejiang Chinese Medical University, Hangzhou 311402, China

^#^These authors contributed equally to this work.

* Corresponding Author E-mail: jzzhang@northwestern.edu (J. Zhang); [laijiang.sps@zcmu.edu.cn](mailto:laijiang.sps@zcmu.edu.cn) (L. Jiang); [gangliu.cmitm@xmu.edu.cn](mailto:gangliu.cmitm@xmu.edu.cn) (G. Liu)

**Contents**

**Supplementary Table S1-S2**

**Supplementary Fig. S1-S14**

**Supplementary Tables:**

**Table S1.** Minimum inhibitory concentrations (MIC) and minimum bactericidal concentration (MBC) (μg/ml, as gentamicin equivalent) of various materials against *P. aeruginosa* PAO1, “-” indicates the absence of antibacterial activity.

| **MIC and MBC of materials** | | |
| --- | --- | --- |
| Materials | MIC (μg/ml) | MBC (μg/ml) |
| Free Gen | 2 | 4 |
| GNPs | 2 | 4 |
| MV-GNPs | 2 | 4 |
| BsAb-GNPs | 2 | 2 |
| NPs | - | - |

**Table S2.** Quantification of viable bacteria in lung tissue homogenates after treatments. Data was presented on average (*n* = 6).

| ***P. aeruginosa*-induced lung infection** | | | |
| --- | --- | --- | --- |
| Materials | Initial Bacterial Count (CFU/mouse)  Log (CFU) | Final Bacterial Count (CFU/mouse) | Log (CFU) |
| PBS | 5.00E+06 | 3.63E+07 | 7.56 |
| Free Gen |  | 3.71E+06 | 6.57 |
| GNPs |  | 2.76E+06 | 6.44 |
| MV-GNPs |  | 4.92E+04 | 4.69 |
| BsAb-GNPs |  | 8.03E+03 | 3.90 |
| Sham |  | 6.67E+00 | 0.82 |

**Supplementary Figures:**


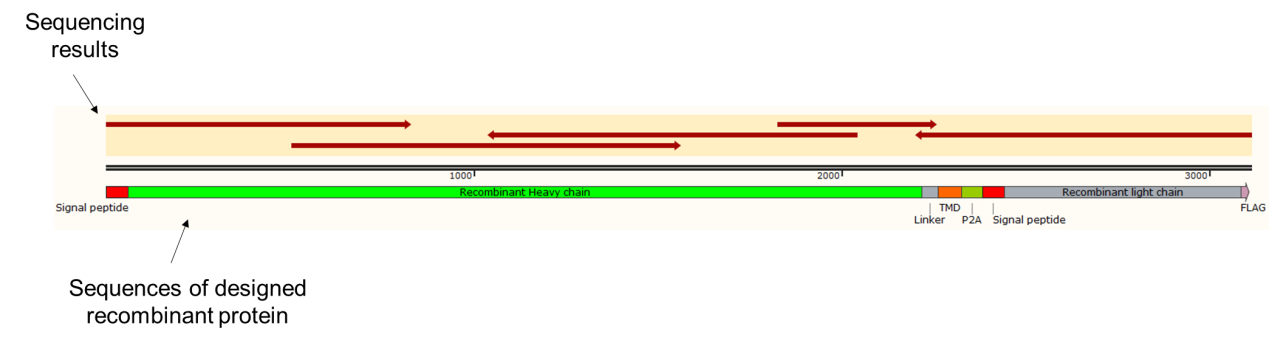


**Fig. S1.** Sequences alignment by the SnapGene software.


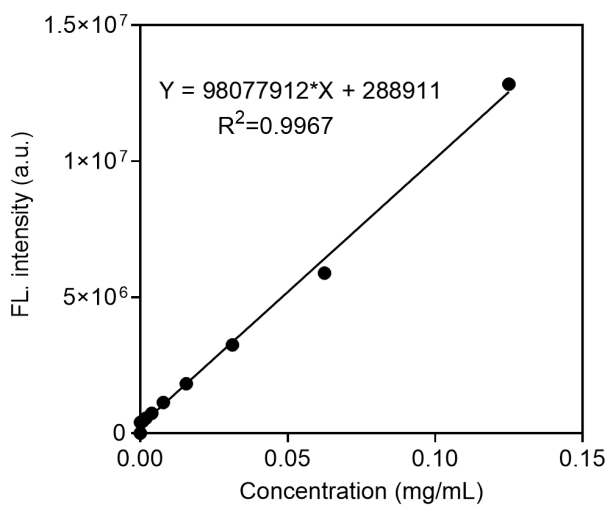


**Fig. S2.** The standard curve for measuring gentamicin using the OPA method.

**
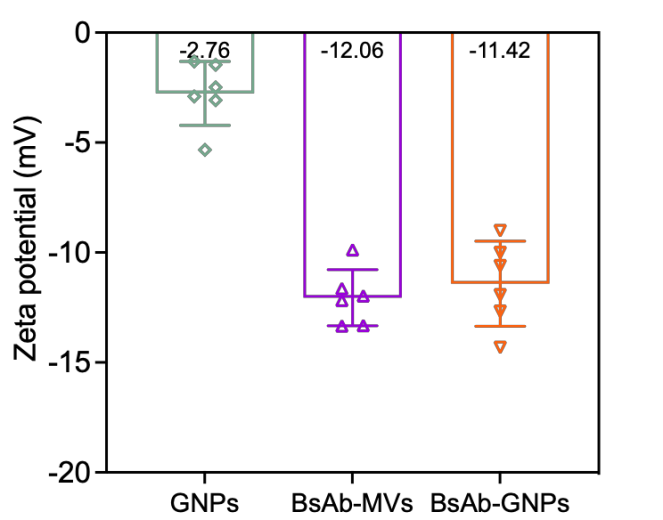
**

**Fig. S3.** The zeta potential of BsAb-MVs, GNPs and BsAb-GNPs.


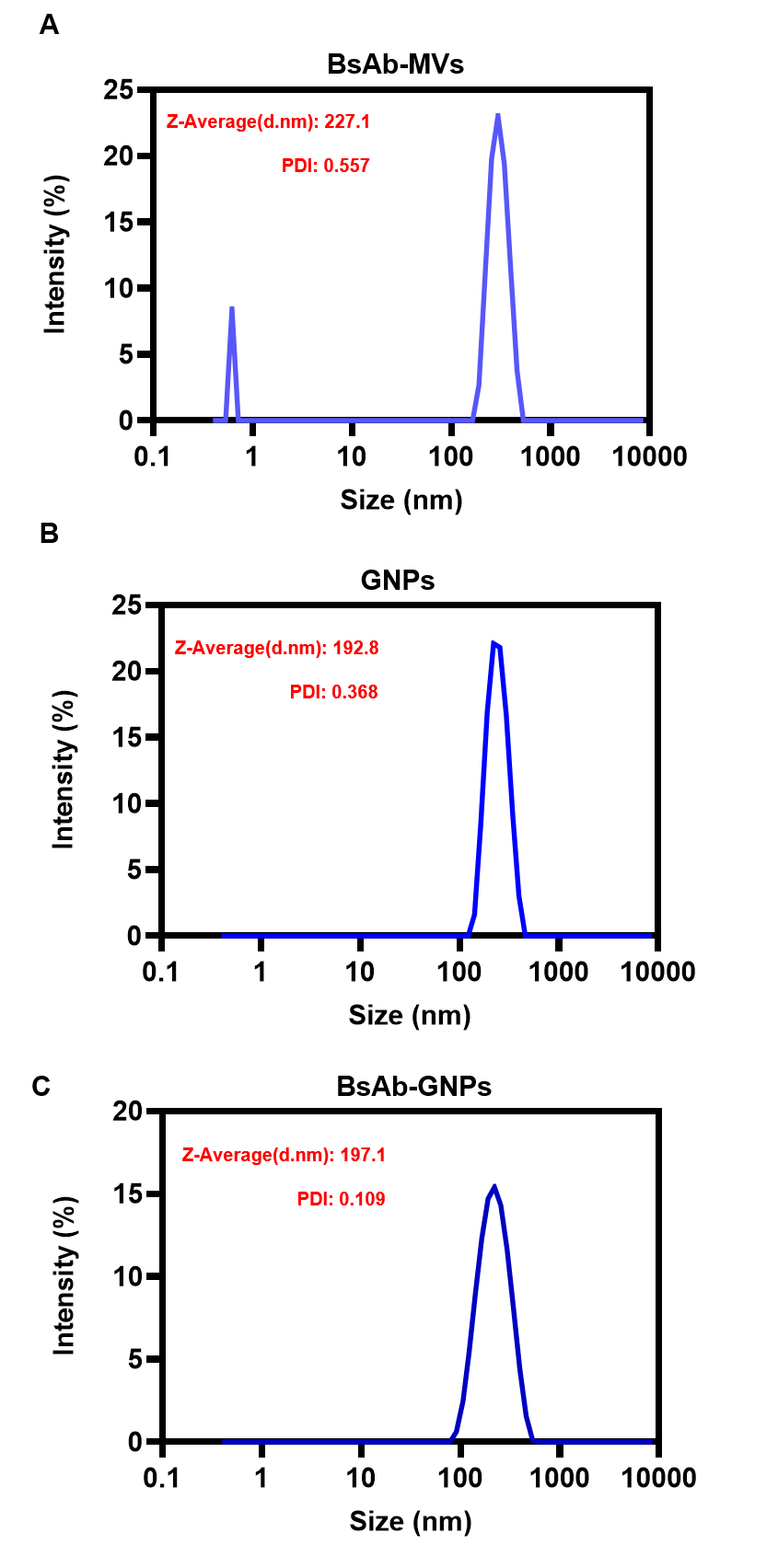


**Fig. S4.** Hydrodynamic radius distribution by intensity and PDI value of (A) BsAb-MVs, (B) GNPs and (C) BsAb-GNPs.


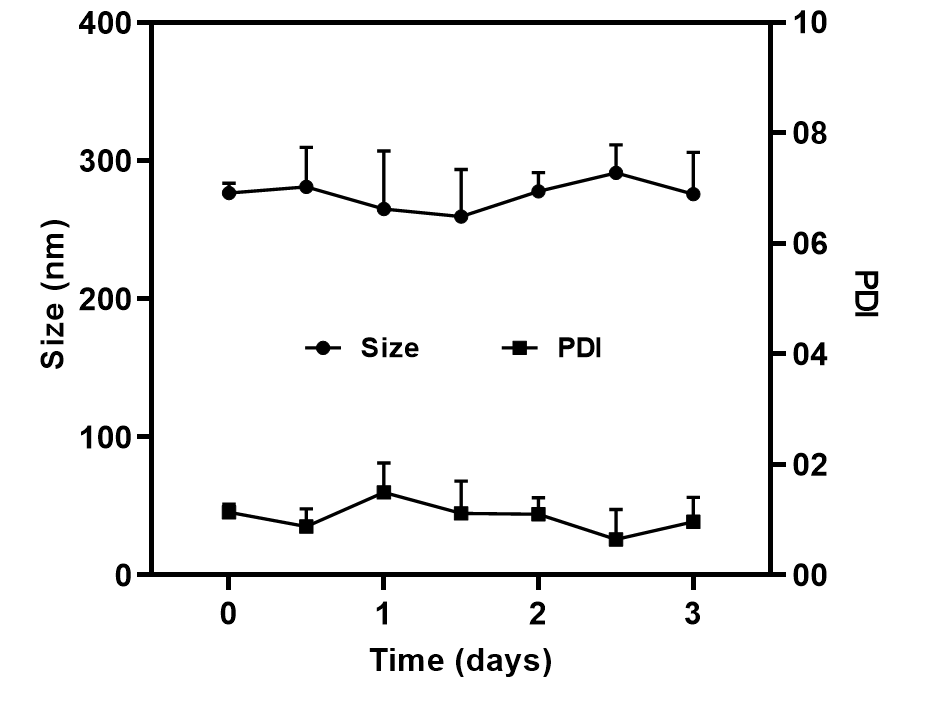


**Fig. S5.** Changes in the size and PDI of BsAb‑GNPs after incubation in 10% serum.


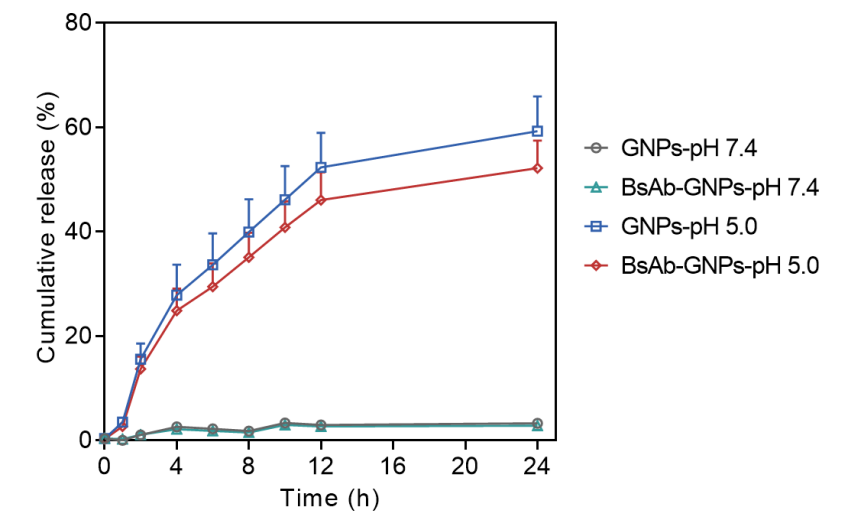


**Fig. S6.** In vitro drug‑release kinetics of GNPs and BsAb‑GNPs under acidic and neutral conditions.


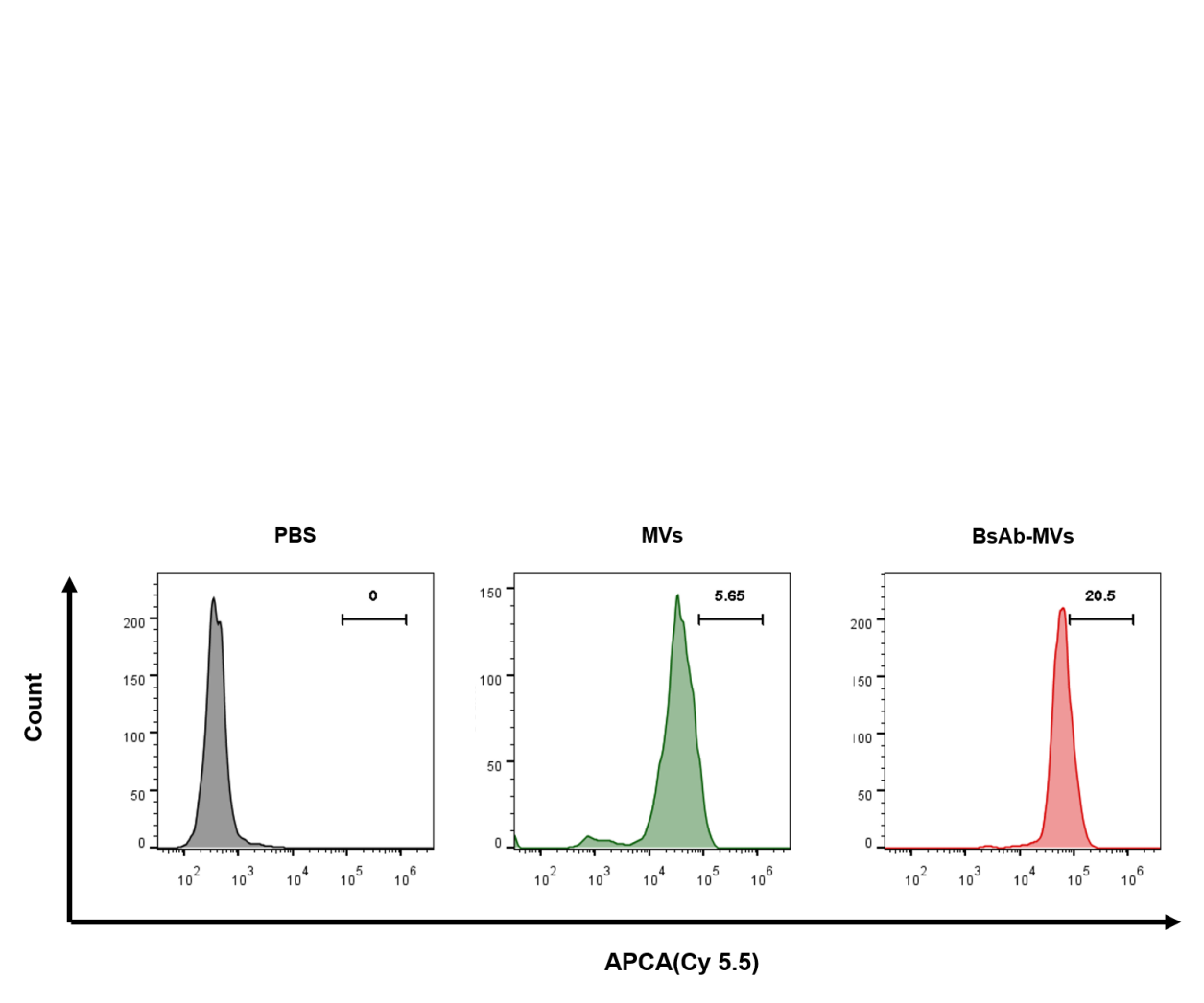


**Fig. S7.** Flow cytometry analysis of BsAb-MVs adhesion to planktonic *P. aeruginosa*.


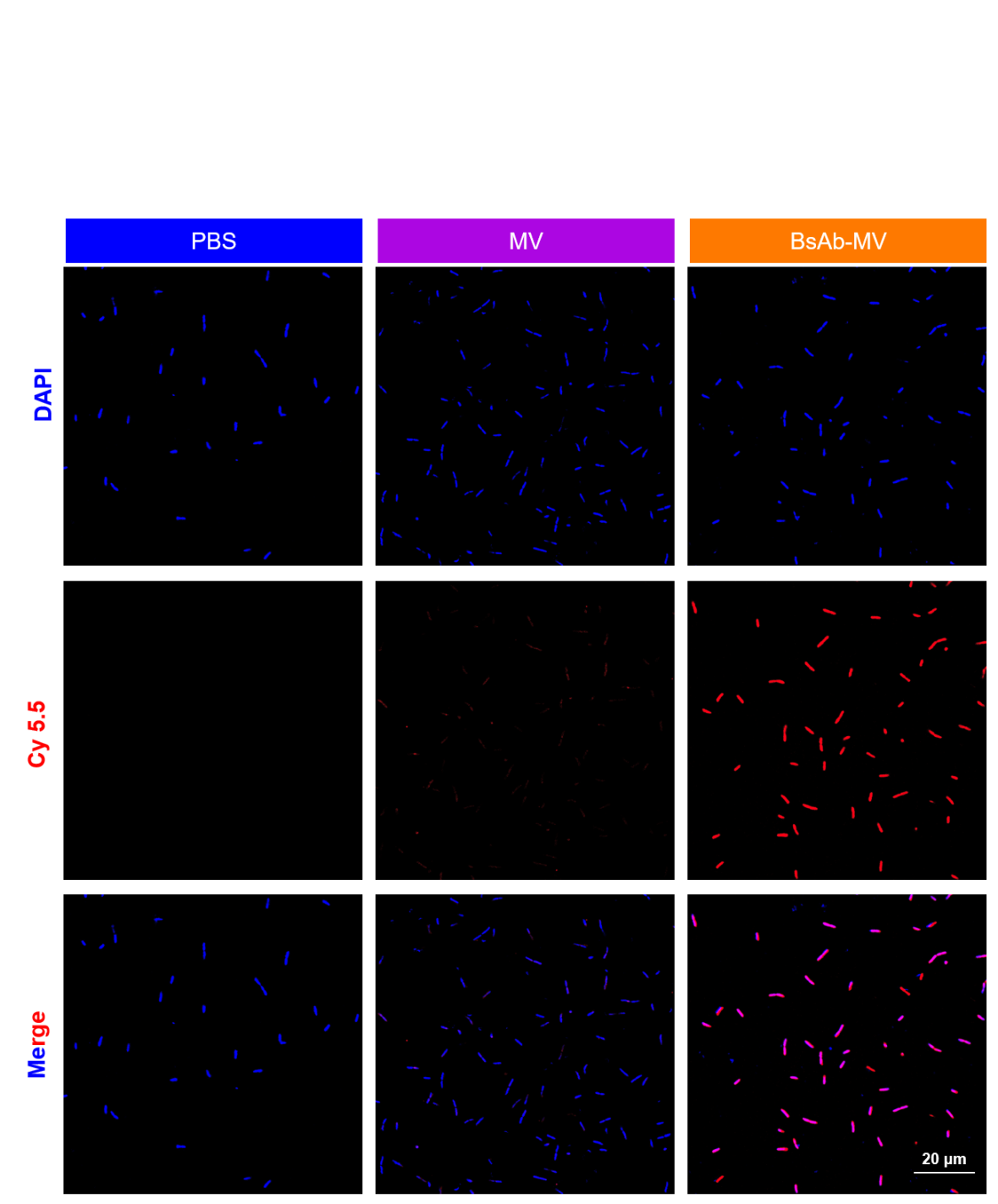


**Fig. S8.** CLSM image of BsAb-MVs adhering to planktonic *P. aeruginosa*, scale bar: 20 μm.


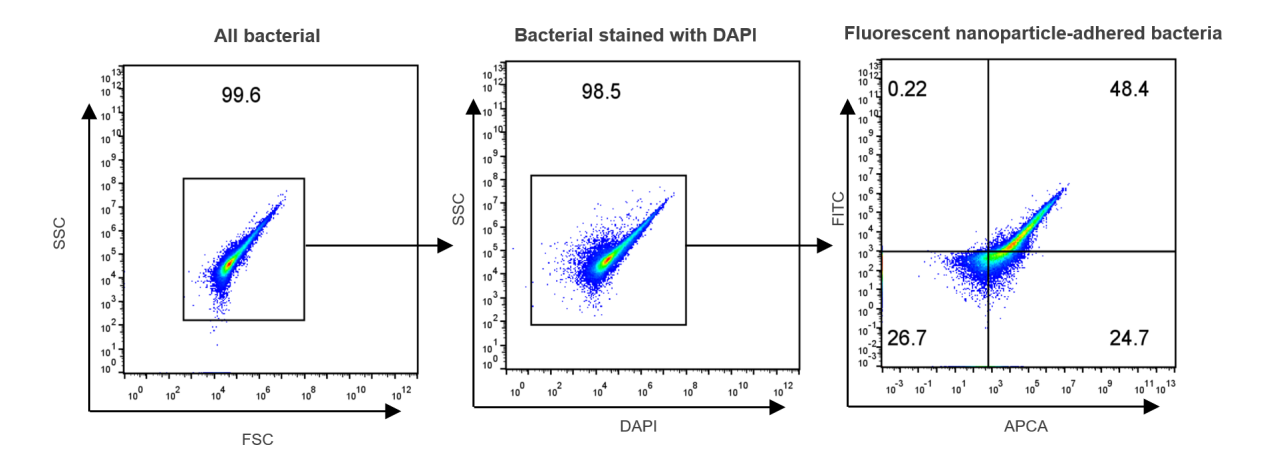


**Fig. S9.** Gating plot for the flow cytometry analysis of BsAb‑NP adhesion to planktonic *P. aeruginosa*, with gating data shown for the BsAb‑NPs group.


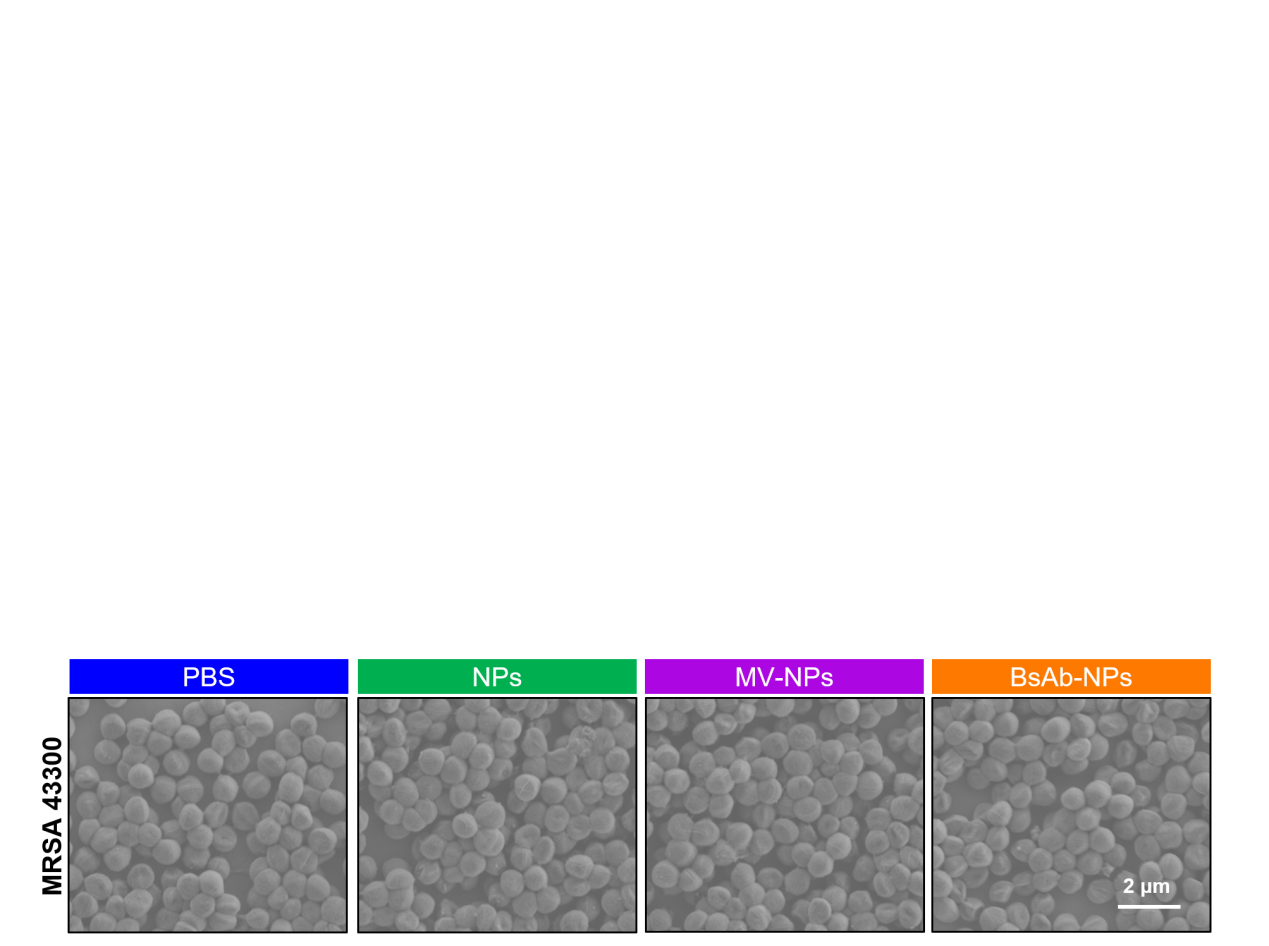


**Fig. S10.** SEM images showing adhesion of BsAb-NPs to planktonic MRSA , scale bar: 2 μm.


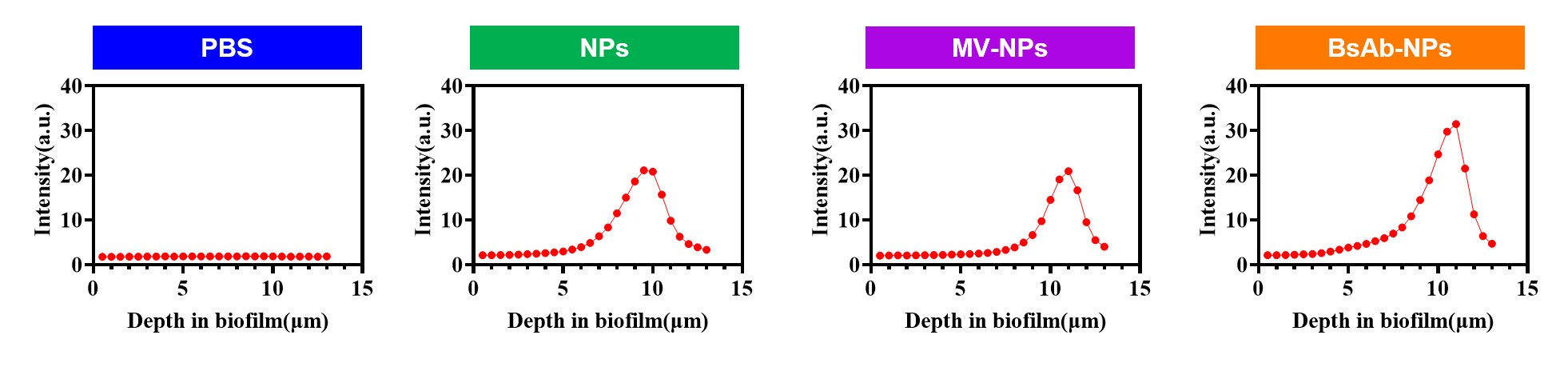


**Fig. S11.** Quantitative fluorescence intensity of fluorescent nanoparticles (NPs, CY5.5) across the biofilm depth obtained from CLSM.


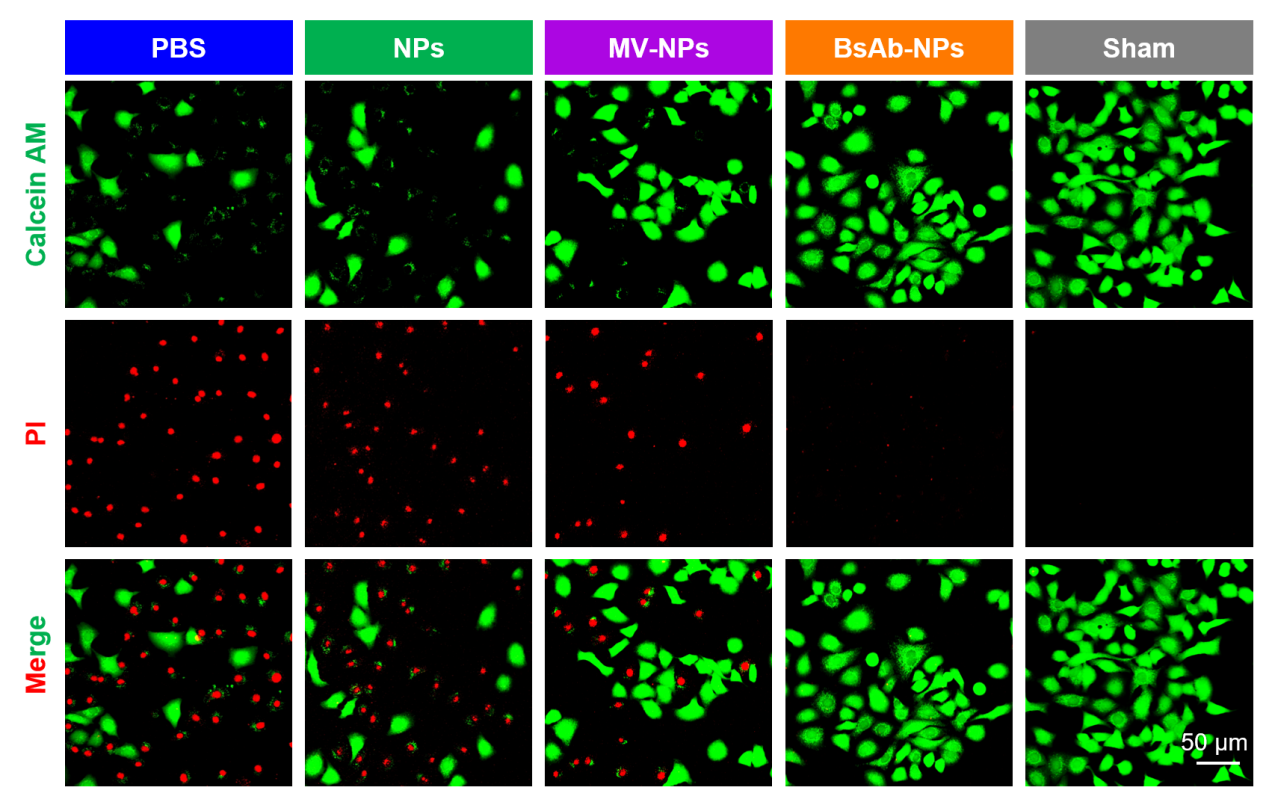


**Fig. S12.** Live/dead images of BsAb-NPs inhibiting *P. aeruginosa* PAO1 invasion of lung epithelial cells. Scale bar: 50 μm, the Sham group represents uninfected cells.


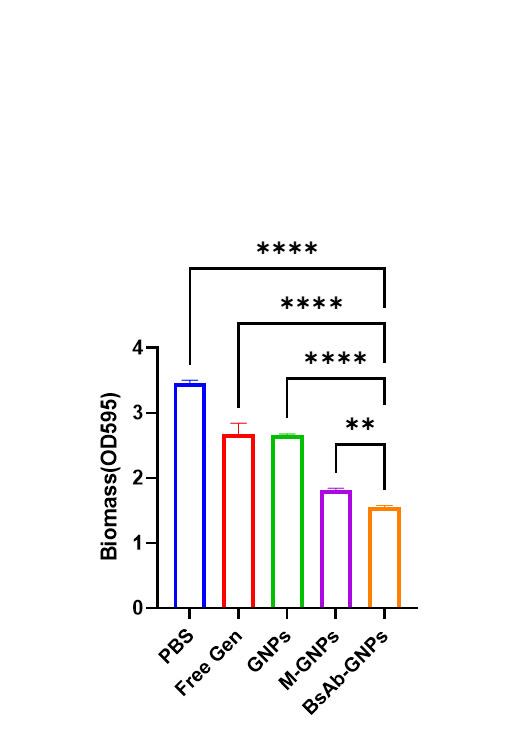


**Fig. S13.** Quantification of residual *P. aeruginosa* biofilm biomass after treatment with different formulations. Statistical analysis was performed using one-way ANOVA followed by Tukey’s post hoc test for multiple comparisons. Statistical significance was defined as ^**^*P* < 0.01 or ^****^*P* < 0.0001.


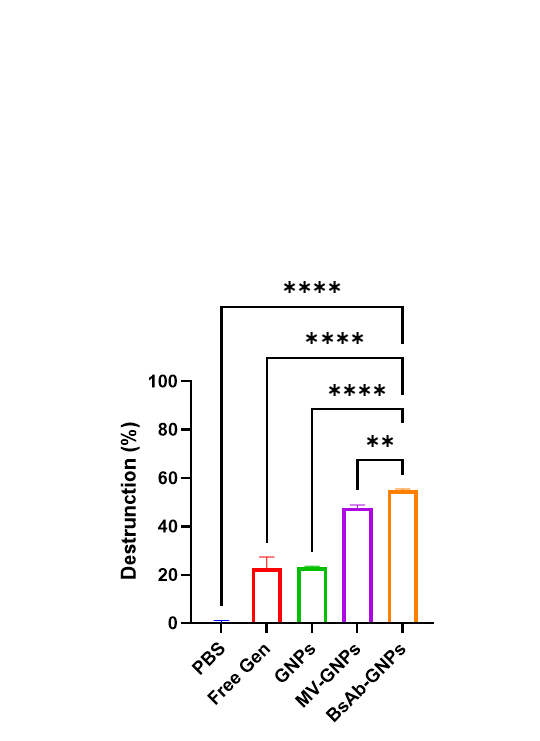


**Fig. S14.** The biofilm destruction rate following treatment with different groups. Statistical analysis was performed using one-way ANOVA followed by Tukey’s post hoc test for multiple comparisons. Statistical significance was defined as ^**^*P* < 0.01 or ^****^*P* < 0.0001.
